# Bioactive Compounds and Antioxidant Capacity of Small Berries

**DOI:** 10.3390/foods9050623

**Published:** 2020-05-13

**Authors:** Michael Zorzi, Francesco Gai, Claudio Medana, Riccardo Aigotti, Sara Morello, Pier Giorgio Peiretti

**Affiliations:** 1 Department of Molecular Biotechnology and Health Sciences, University of Torino, 10125 Torino, Italy; michael.zorzi@unito.it (M.Z.); claudio.medana@unito.it (C.M.); riccardo.aigotti@unito.it (R.A.); 2 Institute of Sciences of Food Production, Italian National Research Council, 10095 Grugliasco, Italy; sara.morello.90@gmail.com (S.M.); piergiorgio.peiretti@ispa.cnr.it (P.G.P.)

**Keywords:** phytochemicals, polyphenols, fatty acid, HPLC-HRMS, berry fruits, antioxidant capacity

## Abstract

The popularity of small berries has rapidly increased in Western countries given their antioxidant, anti-inflammatory, and antimicrobial activities and health-promoting properties. The aim of this study was to compare the fatty acid (FA) profile, phenolic compounds, and antioxidant capacity of extracts of 11 berries cultivated in the North West of Italy. Berry samples were extracted and evaluated for FA profile and total anthocyanin (TAC), total flavonoid contents (TFC), ferric-reducing antioxidant power (FRAP), and for their radical scavenging activities against 2,2′-diphenyl-1-picrylhydrazyl (DPPH^•^) radical. The main polyphenols of berry extracts were characterized by HPLC-DAD-UV-ESI HRMS in positive ion mode. Results showed that the highest TAC and TFC contents were recorded in black currants, blackberries, and blueberries. Maximum and minimum DPPH^•^ radical scavenging activities, Trolox Equivalent Antioxidant Capacity, and FRAP measurements confirmed the same trend recorded for TAC and TFC values. HPLC-HRMS analyses highlight how blueberries and blackberries have the highest concentration in polyphenols. Palmitic, stearic, oleic, linoleic, α-linolenic, and γ-linolenic acids significantly differ between berries, with oleic and α-linolenic acid representing the most abundant FAs in raspberries. Among the berries investigated, results of phytochemical characterization suggest choosing black currants and blueberries as an excellent source of natural antioxidants for food and health purposes.

## 1. Introduction

Due to their antioxidant, anti-inflammatory, and antimicrobial activities [1], the popularity of small berries has rapidly increased in Western countries given their composition and health-promoting properties related to the prevention or the onset delay of chronic age-related diseases [2]. These beneficial effects are provided by a structurally varied range of bioactive compounds, such as dietary phenolics [3].

Polyphenolic compounds can be found in a wide range of foodstuffs, such as tea, red wine, coffee, olives, and dark chocolate, but these analytes can be found also in large quantities in small berries [4,5]. These compounds can be divided into different classes, of which the four major classes of polyphenols are flavonoids, lignans, phenolic acids, and stilbenes. Fruits like berries are particularly rich in flavonoids [6].

Increased consumer interest in berry health-promoting effects has been recorded in Italy, particularly in the North-Western regions, where berry farmers have more than tripled the extension of cultivated areas compared with the last decade. Currently, at a regional level, 80% of berry production is dedicated to blueberries (*Vaccinium corymbosum*) and 14% to raspberries (*Rubus idaeus*), while the remaining portion is dedicated to the cultivation of black currants (*Ribes nigrum*), red currants (*Ribes rubrum*), white currants (*Ribes pallidum*), blackberry (*Rubus fruticosus*), white and red gooseberries (*Ribes grossularia* L.), and, more recently, goji (*Lycium barbarum* L.) [7]. Moreover, these valuable fruits can be grown in lands otherwise abandoned due to poor productivity [8].

The aim of this study was to compare the bioactive compounds and antioxidant capacity of extracts of different cultivated berry fruits harvested during the 2016 summer season in the Piedmont Region (NW Italy). The phytochemical characterization of each sample will provide useful knowledge about berries with the highest bioactive compounds that could be used as both a natural antioxidant for fresh consumption and a preservative for food processing.

## 2. Materials and Methods

### 2.1. Plant Material and Chemicals

Raspberries, black currants, red currants, white currants, white and red gooseberries, blackberry, goji, and three Cv. (Duke, Blue Ray, and Misty) of blueberries were purchased from local farms in the Piedmont Region (Table 1). Berries were harvested manually at the appropriate maturity during the 2016 growing season. Two repetitions were sampled per berries (*m* = 250 g/repetition). Upon arrival in the laboratory, berries were freeze-dried using a laboratory lyophilizer (5 Pascal, Trezzano sul Naviglio, Italy) and then finely ground and vacuum-packed and stored at −20 °C for analysis. Dry matter (DM) was determined in triplicate by freeze-drying each berry sample in a laboratory lyophilizer at −50 °C to a constant weight.

The standards (+)-catechin (>95% purity), quercetin (>95% purity), kaempferol (>90% purity), chlorogenic acid, cyanidin, cyanidin 3-glucoside chloride, delphinidin 3-galactoside chloride, and kaempferol 3-O-β–rutinoside were purchased from Sigma-Aldrich (Milan, Italy). All solvents and reagents utilized were from VWR Italia (Milan, Italy). Sodium persulfate, ferrous chloride, the Folin–Ciocalteau phenol reagent, 2,2′-azinobis-(3-ethylbenzothiazoline-6-sulfonic acid) (ABTS), 2,2′-diphenyl-1-picrylhydrazyl (DPPH^•^), 2,4,6-tri(2-pyridyl)-s-triazine (TPTZ), and 6-hydroxy-2,5,7,8-tetramethyl-chroman-2-carboxylic acid (Trolox) were purchased from Sigma-Aldrich (Milan, Italy). All the aqueous solutions were prepared with ultrapure water (Millipore MilliQ™, Bedford, MA, USA).

### 2.2. Sample Preparation 

Samples weighing 2 g were extracted with 10 mL of 80:20 (*v*/*v*) methanol:water with formic acid (1%) using a Polytron tissue homogenizer (Type PT 10–35; Kinematica GmbH, Luzern, Switzerland) for 1 min and centrifuged for 20 min at 4000× *g* [9]. The pellets were re-extracted, and supernatants were pooled and then evaporated under vacuum at room temperature using a Speedvac (SC210A; Savant Instruments, Farmingdale, NY, USA). The residues were re-suspended in 10 mL of methanol/formic acid (99:1, *v*/*v*). Aliquots were stored at −80 °C before analysis. All analyses were performed in triplicate. Using external calibration curves based on quercetin and kaempferol analytes (which are always detectable in all the samples), the content of polyphenolics in the extracted sample averaged 15 mg/kg.

### 2.3. Total Anthocyanins Content 

Total monomeric plus polymerized anthocyanins in the extracts were estimated spectrophotometrically using the pH single method [10]. The extracts were diluted with two buffer solutions of pH 1 and 4.5. The absorbance of each dilution was measured at 510 nm (A510) and 700 nm (A700) against a distilled water control. The total anthocyanin concentration was obtained from the equation:

C_tot_(mg/L) = (A_tot_ × MW × DF × 1000)/ε × CPL

where absorbance *A_tot_* is calculated as A510–A700, *ε* is the molar absorbance coefficient of cyanidin-3-glucoside, *MW* is the molecular weight of cyanidin-3-glucoside (449.2 g/mol), *DF* is the dilution factor, and *CPL* is the cell path length (1 cm). Total anthocyanins content was expressed in two ways: as mg of cyanidin-3-glucoside equivalents per g of extract and as mg of cyanidin-3-glucoside equivalents per g fresh matter.

### 2.4. Total Flavonoids Content 

The total flavonoids content was measured using a colorimetric assay [11]. An aliquot (1 mL) of extracts (concentration 1 mg/mL) was added to a 10 mL volumetric flask containing 4 mL of deionized H_2_O. To the flask, 0.3 mL of 5% NaNO_2_ was added and 0.3 mL of 10% AlCl_3_ 5 min later. After 6 min, 2 mL of 1 M NaOH solution was added and the total volume was made up to 10 mL with deionized H_2_O. The solution was mixed well, and the absorbance was measured at 510 nm against reagent control containing distilled water instead of the extract. The total flavonoid content was expressed in two ways: as mg of rutin equivalents per g of extract and as mg of rutin equivalents per g of fresh matter.

### 2.5. Trolox Equivalent Antioxidant Capacity (TEAC)

The TEAC was determined using a colorimetric method [12]. For this assay, 2,2′-azino-bis(3-ethylbenzothiazoline-6-sulfonic acid) cation radical (ABTS^•+^) solution was prepared by dissolving 96 mg of ABTS in 2.45 mM Na_2_S_2_O_8_. This mixture was shaken for 16 h at room temperature in the dark until reaching a stable oxidative state. Before analysis, the ABTS^•+^ stock solution was diluted with methanol to an absorbance of 0.70 ± 0.02 at 734 nm. For the spectrophotometric assay, 2 mL of the ABTS^•+^ solution and 20 μL of extracts were mixed and the absorbance was recorded at 734 nm with the spectrophotometer DU 7500 (Beckman Instruments Inc., Fullerton, CA, USA) after sample incubation at 30 °C for 6 min (TH-24 block heater; Meditherm, Poland). The calibration curve was plotted using 6-hydroxy-2,5,7,8-tetramethylchromane-2- carboxylic acid (Trolox) as a standard. The results were expressed as mmol Trolox equivalents per g of extract and as mmol Trolox equivalents per g of fresh matter.

### 2.6. Ferric-Reducing Antioxidant Power (FRAP)

The FRAP assay was performed using a colorimetric method [13]. The sample solution analyzed was first properly diluted with deionized water to fit within the linearity range. The working FRAP reagent was prepared by mixing 10 volumes of 0.3 M acetate buffer, pH 3.6, with 1 volume of 10 mM 2,4,6-tris(2- pyridyl)-S-triazine in 40 mM HCl and with 1 volume of 20 mM FeCl_3_ 6 H_2_O. A volume of 2.25 mL of a working FRAP reagent was warmed to 37 °C. Then, 75 μL of the sample and 225 μL of deionized water were added to the FRAP reagent and the absorbance was taken at 593 nm against the reagent blank after 30 min of incubation with the spectrophotometer DU 7500 (Beckman Instruments Inc., Fullerton, CA, USA). The FRAP value was calculated and expressed as mmol Fe^2+^ equivalents per g of extract and as mmol Fe^2+^ equivalents per g of fresh matter (using the calibration curve prepared for Fe_2_SO_4_).

### 2.7. Scavenging of the DPPH^•^ Radical 

The scavenging effect of phenolics from the extracts was monitored as described in the literature [14]. A 0.1 mL methanolic solution containing between 0.2–0.8 mg/mL of extract was mixed with 1 mL of deionized water and then added to a methanolic solution of DPPH^•^ (1 mM, 0.125 mL). The mixture was vortexed for 1 min, left to stand at room temperature for 20 min in the dark, and absorbance of the solution was then measured at 517 nm with the spectrophotometer DU 7500 (Beckman Instruments Inc., Fullerton, CA, USA).

The data were expressed as EC_50_ values. This value, under the experimental conditions employed during analysis, is linked to the concentration of sample required for 50% scavenging of DPPH^•^ radical (expressed as mg/mL).

### 2.8. HPLC-DAD-UV-ESI HRMS Analytical Method 

All the extracts were characterized by HPLC-DAD-UV-ESI HRMS in positive ion mode. Based on the information obtained from the UV-VIS spectrum and of the accurate mass of precursor ions and tandem MS experiments, the main polyphenols were identified and quantified.

The instrument setup for the polyphenols (phenolic acids, flavanols, flavonols, and anthocyanins) analyses consisted of a Dionex Ultimate 3000 HPLC system equipped with a DAD detector (Thermo Scientific Surveyor, Milan, Italy), coupled with a High-Resolution Mass Spectrometer LTQ-Orbitrap (Thermo Scientific, Milan, Italy) through an ESI interface operating in positive ion mode.

Two different instrumental conditions were adopted to analyze anthocyanins and other flavonoid compounds. The procedure described in the literature [15] with slight modifications was used. For anthocyanin compounds an RP C18 column (Varian Pursuit C18, 150 × 2.0 mm, 3 μm particle size; Agilent, Milan, Italy) at 200 μL/min flow rate was used. The elution solvents adopted were formic acid 0.1% in methanol (B) and in water (A). The gradient profile was 0–6 min from 10% to 15% of B, 6–12 min from 15% to 25% B, 12–16 min from 25% to 30% B, an isocratic step to 30% of solvent B for 14 min, and finally 30–42 min from 30% to 100% B. Injection volume was 20 μL. The tuning parameters used for the ESI source were: capillary temperature 270 °C, flow rate of sheath gas and auxiliary gas set at 35.0 and 15.0 arbitrary units, capillary voltage 8.0 V, source voltage 4.5 kV, and tube lens 65 V. Full scan spectra were acquired in positive ion mode in the range 250–1000 *m/z* with the resolution of 30,000 full-width at half the maximum peak height (FWHM). MS^n^ spectra were acquired in the range between ion trap cut-off and precursor ion *m/z* values.

For other polyphenol compounds (phenolic acids, flavanols, and flavonols) a biphenyl stationary phase (Pinnacle DB BiPh, 150 × 2.1 mm, 3 μm particle size; Resteck, Milan, Italy) at 200 μL/min flow rate was used. The elution solvent adopted was methanol (B) and ammonium acetate 5 mM (A). The gradient profile was 0–3 min to 2% of B, 3–60 min from 2% to 62% B, 60–65 min from 62% to 100% B. The tuning parameters used for the ESI source were: capillary temperature 270 °C, flow rate of sheath gas and auxiliary gas set at 35.0 and 15.0 arbitrary units, capillary voltage 16.0 V, source voltage 3.5 kV, and tube lens 55 V. Full scan spectra were acquired in positive ion mode in the range 100–1000 *m/z* with the resolution of 30,000 (FWHM). MS^n^ spectra were acquired in the range between ion trap cut-off and precursor ion *m/z* values.

The method reveals satisfactory linearity in the range 0.01 (LLOQ)–500 μg/g.

The HPLC technique is the most widely employed analytical instrumentation for the identification and quantification of polyphenols in fruit and vegetable matrices [16]. The identity of the phenolic compounds was investigated by means of the information obtained using the UV-VIS spectra, compared to the online databases and the literature, as well as the accurate mass of precursor ions and tandem MS product ions. The UV-Vis spectra of the compounds were recorded between 200 and 650 nm: for example, flavonols and anthocyanins were monitored at 360 and 520 nm, respectively, and hydroxycinnamic acids at 260 nm.

### 2.9. Fatty Acid Composition by Gas Chromatography 

Fatty acid (FA) composition was determined on the lyophilized samples. The lipid extraction of the samples was performed according to Peiretti et al. [17]. The FA methyl esters in hexane were then injected into a gas chromatograph (GC1000 DPC; Dani Instruments S.p.A., Cologno Monzese, Italy) equipped with a flame ionization detector (FID). The separation of the FA methyl esters was performed using a Famewax™ fused silica capillary column (30 m × 0.25 mm, 0.25 µm particle size) (Restek Corporation, Bellefonte, PA, USA). The peak area was measured using a Dani Data Station DDS 1000. Each peak was identified and quantified because of pure methyl ester standards (Restek Corporation, Bellefonte, PA, USA). All analyses were performed in triplicate.

### 2.10. Statistical Analysis 

The results were analyzed by one-way analysis of variance (ANOVA) using SPSS version 11.5.1 for Windows (SPSS Inc., Chicago, IL, USA). Significant differences were considered at *p* < 0.05. Multiple comparisons of the means were conducted using a post hoc (Duncan test) procedure to establish any differences among berries. For comparison of the results of TAC, TFC, TEAC, FRAP, and DPPH assays, the coefficients of correlation were determined for each combination by a Pearson correlation test.

## 3. Results and Discussion

### 3.1. Total Anthocyanins and Flavonoids Contents 

Results reported in Table 2 on the fresh material show that the highest TAC and TFC values were recorded in black currants. Regarding the TAC and TFC of blackberries and raspberries, Sariburun et al. [18] found that blackberries have a higher value than raspberries in a similar trend to that found in our work.

Regarding TAC, maximum values expressed on the fresh material, which ranged from 5.92 mg/g FM (black currant) to 0.02 mg/g FM (blueberry *Cv*. Blue Ray), were found in blackberry species. The total amount and quality of anthocyanins in black currants and other berries are in general agreement with previous papers [19]. Kähkönen et al. [20] found a TAC content of 2.36 mg/g fresh weight in the phenolic extracts of black currant.

The TFC values expressed on the fresh material showed the highest value in black currants (19.3 mg rutin eq./g), while the lowest values were recorded in raspberries (1.72 mg rutin eq./g) and goji (0.75 mg rutin eq./g). Similar values were found in goji berries by Islam et al. [21] and in blueberries by Brito et al. [22].

### 3.2. Antioxidant Capacity Assays 

Results of different antioxidant capacity assays (TEAC, FRAP, and DPPH) performed are reported in Table 3. 

Goji fruits have the lowest antioxidant capacity compared with black currant, followed by white currants and white gooseberries, which have the highest. Similar trends were observed for all antioxidant capacity assays and confirmed the results obtained for TAC and TFC, as also demonstrated by their correlation coefficients reported in Table 4.

The results of TAC and TFC with three antioxidant capacity assays are significant; their correlation is statistically different from 0, with a significance level α = 0.01 and α = 0.05; they correlated positively with TEAC and FRAP and negatively with EC_50_ values on DPPH^•^. Among antioxidant capacity assays, FRAP resulted in the best correlation, with a correlation coefficient of 0.729 for TAC, while TFC in all assays was the least correlated parameter. Finally, TEAC and DPPH assays showed a similar trend but with the lowest correlation coefficients. Comparing the antioxidant features of six endemic Chilean berries with blueberries, Brito et al. [22] found a similar correlation for TFC with DPPH and FRAP assays. 

**Figure 1:**
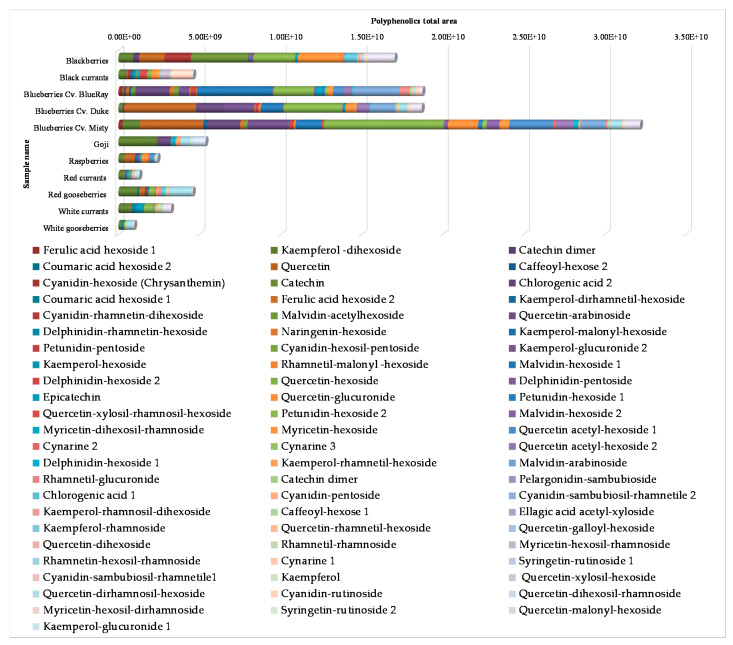
Histogram of the 70 different analytes found in the 11 berry fruits investigated.

FRAP values reported in Table 3 are lower than these values reported in the literature [23,24]. Similar conclusions were reported by Borges et al. [9] in a study carried out on the antioxidant capacity of black currant, blueberry, raspberry, red currant, and cranberry extracts using the FRAP assay. These authors found that the highest antioxidant capacity of black currants and blueberries was due to a major content of anthocyanins, whereas the lowest values of red currants and cranberries were mainly due to a reduced anthocyanin content. Moreover, the structure of anthocyanidins also influences their antioxidant capacity; in fact, cyanidin and delphinidin have higher antioxidant capacity than malvidin, pelargonidin, petunidin, and peonidin [25]. Therefore, in our work, the major antioxidant power detected in black currant fruits could be attributed to their cyanidin and delphinidin content (Figure 1 and Table S1).

Regarding berry anthocyanins, Kähkönen et al. [20] found that those of black currant were highly active radical scavengers and effective antioxidants utilizing the DPPH test and human low-density lipoprotein in vitro assays. 

Another important contribution to the berry antioxidant capacity is due to their content of flavonols, which are considered more powerful antioxidants than anthocyanins [26]. 

### 3.3. HPLC-DAD-ESI HRMS 

The berry extracts were classified by the content of different polyphenolic classes. The results showed that the berries with the highest and lowest polyphenol abundance were blueberries (*Cv*. Misty) and white gooseberries, respectively. Indeed, within the berry family, blueberries and blackberries contain high quantities of antioxidant species, such as anthocyanin [27,28]. 

Table S2 reports the 70 different compounds identified or tentatively identified, their [M + H]^+^, the occurrence in different samples, and the most important MS/MS fragments, shown in order of decreasing intensity.

To clarify the relative distribution percentage of polyphenolic compounds in each berry, a pie chart for each berry fruit is reported (Figure S1).

Most of these compounds belong to the flavonol class: mono and di-glycosylated compounds of myricetin, kaempferol, and quercetin are the most abundant. As reported in the literature [28], flavonols such as quercetin, myricetin, and kaempferol and their aglycons are the most abundant in foods. The glycosides quercetin species represent, among all of the detected molecules, the highest percentage among the flavonols family in all of the berries analyzed [29]. Many of these phytochemical aglycon compounds detected exist as mono-, di- and ter-glycosidic polyphenols and the sugar units are linked in different positions on the skeletons of the molecules (Table S1). The presence of these analytes is known: the flavonols and their glycosylated analogues are potent antioxidants that the plants synthetize to protect themselves from reactive oxygen species [30].

Glucose, galactose, and arabinose are the most frequently occurring sugars bounded to polyphenol species to generate the different aglycons [29]. In contrast, if we consider other analytes belonging to the phenolic acid and coumarin families, they are present in lower concentration and not in all berries.

Regarding anthocyanin classes, anthocyanidins of cyanidin, delphinidin, malvidin, and petunidin were recorded in all berries with the exceptions of goji, white currant, and white gooseberry. Among fruits, berries are one of the richest sources of these analytes in nature [23] and results are consistent with previous research [29]: white currant is one of the berries with the lowest number of total flavonol molecules.

Cyanidin was the most commonly occurring anthocyanidin found in five fruits (blackberry, black currant, raspberry, red currant, and red gooseberry), meanwhile, malvidin and petunidin glycosides were detected the most in blueberries. Delphinidin glycosides were the prevailing form of anthocyanins in black currant and blueberry fruits.

These results are confirmed by Lee et al. [31]. These authors identified different anthocyanins, such as delphinidin-3-O-galactoside, delphinidin-3-O-glucoside, petunidin-3-O-glucoside, and malvidin-3-O-galactoside, in blueberries, and delphinidin-3-O-rutinoside and cyanidin-3-O-rutinoside as the major anthocyanin species in black currant. 

However, it is important to remember that the polyphenol concentrations among the berries, and foods in general, can greatly differ depending on various factors such as genetic composition, technology, season, cultivar species, and growing location [27].

Finally, a schematic histogram of the data obtained is reported in Figure 1. Each chromatogram bar represents the single berry analyzed. The height of each bar is instead provided by the sum of all the areas of the chromatographic peaks of the polyphenols found for each single species. This graphical view shows clearly how the berries with the highest concentrations in polyphenolic compounds are blueberries and blackberries [27,28]. On the contrary, white gooseberry and red currant samples are those with the lowest polyphenolic amount. Finally, all the results found are in accordance with Skenderidis et al. [32].

### 3.4. Fatty Acid Composition 

The fatty acid profiles, saturated fatty acids (SFA), monounsaturated fatty acids (MUFA), polyunsaturated fatty acids (PUFA), and the *n*-6/*n*−3 PUFA ratio of the studied berries are described in Table 5. The most abundant FAs present in all studied berries were palmitic, heptadecanoic, stearic, oleic, linoleic, and α-linolenic acid, with some minor FAs, such as palmitoleic, γ-linolenic, arachidic, and lignoceric acid, that are not present in all samples. Palmitic, stearic, oleic, linoleic, α-linolenic, and γ-linolenic acids significantly differ between berries; in particular, palmitic and stearic acid were most abundant in black currants, while goji berries contained the lowest amount of these two FAs. Oleic and α-linolenic acid were the most abundant in raspberries, while the currants did not contain oleic acid, and goji berries contained the lowest amount of α-linolenic acid. Linoleic acid was the main FA in goji berries while the lowest content was found in raspberries.

The three currant species samples were found to have the highest levels of SFA ranging between 61.15 and 74.25 g/100 g. As reported previously, the goji sample had the lowest percentage of 18.67 g/100 g. Independently from the sample considered, stearic acid is the most abundant SFA species.

On the contrary, focusing on the PUFA group, linoleic acid was the most abundant polyunsaturated fatty acid in berry fruits, ranging from 9.15 g/100 g of the raspberry to the surprising 54.05 g/100 g of the goji berry sample. This result clearly demonstrates that the goji sample is a berry rich in polyunsaturated fatty acids.

Regarding SFA, MUFA, and PUFA content, all parameters were significantly different between all berries. Black currants showed the highest content of SFA with the lowest in goji berries. MUFA content was the highest in raspberries and the lowest in black currants. The highest amount of PUFA was found in goji berries, while the lowest amount was found in black currants. Regarding the *n*−6/*n*−3 PUFA ratio, raspberries showed the lowest ratio (0.41), while in goji berries this ratio amounted to 8.26. It is also important to note that the FA profile of whole berries has not yet been reported. Similarly, for goji berries, only limited literature data are available.

Considering the MUFA group, the values ranged from 2.50 g/100 g of black currant to 22.76 g/100 g of the raspberry sample. Oleic acid is the most abundant MUFA species among all the samples investigated.

Contrary to what has been reported so far, we can say that each berry fruit has a different composition in terms of fatty acids. Blackberry, the three *Cv.* Blueberries, and the three different currants are abundant in SFA and PUFA, and goji is rich in PUFA fatty acids; regarding MUFA fatty acids, the content is less than 15 g/100 g in all berries, with the exception of goji (18.23 g/100 g) and raspberry (22.72 g/100 g).

All studied berries were a good source of essential FAs and their FA profile may differ from those of the respective seed oils that usually have a high content of PUFA if compared with the whole berry. Manríquez-Torres et al. [33] determined the FA profile of blackberry juice and showed the presence of SFA (myristic and stearic acid), MUFA (oleic acid), and PUFA (linoleic and α-linolenic acid). Moreover, they determined the impact of thermo-ultrasound, compared with pasteurization, on the FA profile of blackberry juice and found that myristic, α-linolenic, oleic, and linoleic acids were not affected by the treatments, except for stearic acid, whose amount was particularly higher in the control than in the pasteurized and thermo-ultrasonicated samples. Parry et al. [34] determined the FA profile of red raspberry (*Rubus ideaus*), black raspberry (*Rubus occidentalis* L.), cranberry (*Vaccinium macrocarpon*), and blueberry (*Vaccinium corymbosum*) among different fruit seed flours. They found that cranberry seed flour showed the highest α-linolenic acid content with a very low *n*−6/*n*−3 PUFA ratio at approximately 1.2, while this ratio ranged from 6.9 to 8.9 in red raspberry seed flour.

Radočaj et al. [35] determined the FA profile of blackberry and raspberry seed oils extracted from fruit processing waste (air and oven dried berry pomace). All investigated oils had high levels of oleic, *n*−6, and *n*−3 PUFA with their desirable ratio. Blackberry oil had much higher oleic and linoleic acid content, while raspberry oil had much higher α-linolenic content and therefore much lower *n*−6/*n*−3 PUFA ratio (1.69–1.86). Van Hoed et al. [36] determined the FA profile of oil obtained from seeds of different berries (blackberry, blueberry, cranberry, red raspberry, strawberry, and kiwifruit) and found high amounts of unsaturated FAs in all seed oil samples (up to 96 g/100 g in red raspberry). When red raspberry and blackberry are compared, differences were observed for all FAs, while in the oils of the cranberry and blueberry only the amounts of α-linolenic and linoleic acid were different. Cranberry contained the highest quantity of MUFA (25 g/100 g) and red raspberry the highest amount of PUFA (85 g/100 g) compared with the other seed oils. Linoleic acid was the most abundant FA, ranging from 37 g/100 g in cranberry to 61 g/100 g in blackberry. All the berry seed oils exhibited a very favorable *n*−6/*n*−3 PUFA ratio, compared with other vegetable oils; in particular, blackberry seed oil showed the highest *n*−6/*n*−3 PUFA ratio (3.58). Yang et al. [37] investigated the composition of oils from the seeds and the soft parts of a range of northern berries extracted by supercritical CO_2_. The seed oils of wild bilberries (*Vaccinium myrtillus* L.), lingonberries (*Vaccinium vitis-idaea* L.), arctic cranberries (*Vaccinium oxycoccos* L.), crowberries (*Empetrum nigrum* L.), and cloudberries (*Rubus chamaemorus* L.), and of cultivated raspberries, black currants, and redcurrants, were all rich in linoleic acid (34–55 g/100 g) and α-linolenic acid (29–45 g/100 g). The seed oils of black currant and redcurrant contained γ-linolenic acid and stearidonic acid. The *n*−6/*n*−3 ratio was 1.9 in redcurrant seed oil and 3.7 in black currant seed oil. Bushman et al. [38] found that the oils from the seeds of red raspberry (*Rubus idaeus* L.), black raspberry (*Rubus occidentalis* L.), boysenberry (*Rubus ursinus x idaeus*), blackberry (*Rubus ursinus*), and evergreen blackberry (*Rubus laciniatus* Willd) contained mainly linoleic acid (52–63 g/100 g), α-linolenic acid (15–31 g/100 g), and 3−8 g 100/g SFA. The red and black raspberry seeds showed higher percentages of oil, the highest amounts of α-linolenic acid, and the lowest amounts of SFA. Furthermore, these authors found less common FAs, such as C18:1n11 and C20:0, present in all five berry seeds, and γ-linolenic acid present only in the two raspberries seeds. Parry and Yu [39] reported that blackberry seed oil is an excellent natural source of linoleic acid (55–58 g/100 g) and α-linolenic acid (35 g/100 g), with a *n*−6/*n*−3 PUFA ratio ranging from 1.6 to 1.7, similar to that found in the present study for blackberries. Parry et al. [40] evaluated the FA profiles of seed oils of blueberry (*Vaccinium corymbosum*) and of three cranberries (*Rubus spp.* L.): red raspberry, boysenberry, and marionberry. They found that the most prevalent FAs in these seed oils were linoleic acid (from 43.5 to 62.8 g/100 g) and α-linolenic acid (from 15.8 to 32.4 g/100 g), with a *n*−6/*n*−3 PUFA ratio that ranged from 1.64 to 3.99. 

The World Health Organization [41] focuses on the ratios of PUFA/SFA and *n*−6/*n*−3 in order to monitor human health status. The WHO advises that the PUFA/SFA ratio should be above 0.4–0.5, while the *n*−6/*n*−3 PUFA ratio should be 4:1. For some of the samples analyzed (red currant, white currant, black currant, red gooseberry, and white gooseberry) the PUFA/SFA ratio falls within the desired range. For the other samples, the PUFA/SFA ratio is always greater than 0.5 but always less than 1.02.

## 4. Conclusions

Evaluation of the composition of the antioxidant compounds in 11 different berry fruits showed that the highest values were recorded in black currants, blackberries, and blueberries. Maximum and minimum DPPH^•^ radical scavenging activities, Trolox Equivalent Antioxidant Capacity, and Ferric-Reducing Antioxidant Power measurements showed the same trend. HPLC-HRMS analyses highlighted that blueberries and blackberries have the highest concentration in polyphenols, while the berry with the lowest polyphenol abundance is the white gooseberry. Palmitic, stearic, oleic, linoleic, α-linolenic, and γ-linolenic acids significantly differ among berries, with oleic and α-linolenic acids representing the most abundant FAs in raspberries, while the berries with the lowest PUFA content are black and white currants.

In conclusion, among the berries investigated, results of phytochemical characterization suggest choosing black currants and blueberries as the best natural antioxidants for food and health purposes. A future aim, thanks to the development of the HPLC-DAD-ESI HRMS analytical method or new methods (e.g., phloroglucinolysis or vanillin test), will be the identification of many other analytes from heterogenous polyphenolic classes (e.g., proanthocyanidin compounds from the tannin group) that were not taken into account in this work.

## Figures and Tables

**Table 1:** Scientific name, area of cultivation, dry matter (DM, g/kg fresh matter) and extract yield of the berries.

Sample	Scientific Name	Latitude	Longitude	DM	Yield ^1^
Blackberry *Cv*. Loch Tay	*Rubus fruticosus*	45°35′ N	8°04′ E	284.5	378.1
Black currants	*Ribes nigrum*	45°35′ N	8°04′ E	753.5	241.9
Blueberries *Cv*. Blue Ray	*Vaccinium corymbosum*	45°35′ N	8°04′ E	498.8	264.5
Blueberries *Cv*. Duke	*Vaccinium corymbosum*	45°03′ N	7°30′ E	589.0	197.7
Blueberries *Cv*. Misty	*Vaccinium corymbosum*	45°35′ N	8°04′ E	473.2	366.5
Goji	*Lycium barbarum* L.	45°04′ N	7°43′ E	266.3	769.9
Raspberries	*Rubus idaeus*	45°03′ N	7°30′ E	249.2	451.6
Red currants	*Ribes rubrum*	44°32′ N	7°65′ E	655.3	162.6
Red gooseberries	*Ribes grossularia* L.	45°03′ N	7°30′ E	691.1	186.9
White currants	*Ribes pallidum*	44°82′ N	7°97′ E	655.7	178.1
White gooseberries	*Ribes grossularia* L.	44°79′ N	7°37′ E	866.4	120.2

^1^ Extract yields expressed as milligrams of extract per gram (dry weight) of fruits.

**Table 2:** Total anthocyanins (TAC; mg cia-3-glu eq./g) and total flavonoids (TFC; mg rutin eq./g) of the berry extracts and fresh material (FM).

Sample	TAC		TFC	
	g Extract	g FM	g Extract	g FM
Blackberry	2.10 ^b^	0.57 ^b^	24.00 ^a^	6.54 ^cd^
Black currants	7.59 ^a^	5.92 ^a^	24.78 ^a^	19.34 ^a^
Blueberries *Cv*. Blue Ray	0.03 ^d^	0.02 ^d^	24.73 ^a^	12.24 ^b^
Blueberries *Cv*. Duke	0.07 ^d^	0.03 ^d^	18.84 ^a^	10.10 ^bc^
Blueberries *Cv*. Misty	0.66 ^c^	0.30 ^c^	24.67 ^a^	11.11 ^b^
Raspberries	0.20 ^d^	0.05 ^d^	6.48 ^b^	1.72 ^e^
Goji	nd ^1^	nd	2.78 ^b^	0.75 ^e^
Red currants	nd	nd	4.03 ^b^	2.78 ^de^
Red gooseberries	nd	nd	8.32 ^b^	5.74 ^d^
White currants	nd	nd	4.54 ^b^	3.12 ^de^
White gooseberries	nd	nd	7.06 ^b^	6.19 ^d^

^a,b,c,d,e^ Values in the column with different superscript letters are significantly different (*p* < 0.05). ^1^ nd = not detected.

**Table 3:** Trolox equivalent antioxidant capacity (TEAC; mmol Trolox eq./g), ferric-reducing antioxidant power (FRAP; mmol Fe^2+^/g), and radical scavenging activities against 2,2′-diphenyl-1-picrylhydrazyl (DPPH^•^) radical DPPH of the berry extracts and fresh material (FM).

Sample	TEAC		FRAP		DPPH
	g Extract	g FM	g Extract	g FM	mg/mL
Blackberry	10.25 ^ab^	2.94 ^de^	7.02 ^b^	2.01 ^cd^	0.28 ^e^
Black currants	12.09 ^a^	9.00 ^a^	10.29 ^a^	7.72 ^a^	0.20 ^e^
Blueberries *Cv*. Blue Ray	7.58 ^bcd^	3.78 ^cd^	4.61 ^cd^	2.57 ^c^	0.48 ^cd^
Blueberries *Cv*. Duke	8.79 ^abc^	5.21 ^bc^	4.78 ^cd^	2.61 ^c^	0.50 ^cd^
Blueberries *Cv*. Misty	6.09 ^cde^	2.87 ^de^	4.18 ^de^	1.98 ^cd^	0.57 ^c^
Goji	2.79 ^e^	0.74 ^e^	1.46 ^g^	0.39 ^f^	1.18 ^a^
Raspberries	4.38 ^de^	1.08 ^e^	3.08 ^ef^	0.76 ^ef^	0.60 ^c^
Red currants	9.39 ^abc^	6.17 ^b^	3.95 ^de^	2.56 ^c^	0.35 ^de^
Red gooseberries	3.47 ^e^	2.40 ^de^	2.02 ^fg^	1.40 ^de^	0.96 ^b^
White currants	9.08 ^abc^	5.96 ^bc^	5.87 ^bc^	3.85 ^b^	0.26 ^e^
White gooseberries	8.23 ^bc^	7.12 ^ab^	4.53 ^cd^	3.92 ^b^	0.49 ^cd^

^a,b,c,d,e,f,g^ Values in the column with different superscript letters are significantly different (*p* < 0.05).

**Table 4:** Correlation coefficients of total anthocyanin content (TAC) and total flavonoids (TFC) and each antioxidant activity assay.

	TEAC ^1^	FRAP ^2^	DPPH ^3^
TAC	0.580 **	0.729 **	−0.338 *
TFC	0.424 **	0.567 **	−0.333 *

^1^ TEAC; Trolox equivalent antioxidant capacity; ^2^ FRAP; ferric-reducing antioxidant power; ^3^ DPPH. * Correlation is significant at the 0.05 level (2-tailed). ** Correlation is significant at the 0.01 level (2-tailed).

**Table 5:** Fatty acid composition of the berries (g/100 g total fatty acid).

Sample	C_16:0_	C_16:1n9_	C_17:0_	C_18:0_	C_18:1n9_	C_18:1n11_	C_18:2n6_	C_18:3n6_	C_18:3n3_	C_20:0_	C_24:0_	SFA ^1^	MUFA ^2^	PUFA ^3^	*n*−6/*n*−3
Blackberries	26.40 ^ab^	1.10	2.80	16.87 ^b^	12.02 ^c^	0.13 ^c^	23.48 ^b^	Nd ^4^	17.25 ^bc^	nd	nd	46.05 ^e^	13.23 ^c^	40.72 ^c^	1.36 ^cd^
Black currants	28.65 ^a^	0.55	5.90	38.23 ^a^	1.63 ^f^	0.32 ^c^	14.58 ^cd^	nd	8.68 ^ef^	1.47	nd	74.25 ^a^	2.50 ^e^	23.25 ^f^	1.67 ^bc^
Blueberries *Cv*. Blue Ray	20.40 ^c^	nd	8.28	18.20 ^b^	3.68 ^ef^	1.60 ^abc^	28.75 ^b^	nd	19.18 ^ab^	nd	nd	54.75 ^cd^	4.82 ^de^	40.37 ^c^	2.08 ^b^
Blueberries *Cv*. Duke	22.35 ^bc^	nd	9.95	22.48 ^b^	3.70 ^ef^	1.15 ^bc^	27.05 ^b^	nd	13.33 ^cde^	nd	nd	46.80 ^e^	5.25 ^de^	47.92 ^b^	1.50 ^cd^
Blueberries *Cv*. Misty	22.43 ^bc^	nd	6.38	24.55 ^b^	5.23 ^ef^	1.95 ^ab^	26.25 ^b^	nd	13.15 ^cde^	nd	nd	53.42 ^de^	7.17 ^d^	39.40 ^c^	2.07 ^b^
Goji	12.50 ^d^	0.55	1.63	3.68 ^c^	16.55 ^b^	1.13 ^bc^	54.05 ^a^	1.50	6.83 ^f^	0.63	0.23	18.70 ^f^	18.25 ^b^	62.37 ^a^	8.26 ^a^
Raspberries	22.13 ^bc^	1.08	6.00	17.85 ^b^	21.57 ^a^	0.07 ^c^	9.15 ^d^	nd	22.17 ^a^	nd	nd	46.00 ^e^	22.72 ^a^	31.30 ^de^	0.41 ^e^
Red currants	23.20 ^bc^	0.67	10.65	26.65 ^ab^	7.17 ^de^	3.12 ^a^	16.07 ^c^	nd	11.83 ^de^	0.65	nd	61.15 ^bc^	10.92 ^c^	27.90 ^def^	1.36 ^cd^
Red gooseberries	23.65 ^abc^	nd	13.85	23.53 ^b^	5.85 ^e^	0.35 ^bc^	17.40 ^c^	nd	13.43 ^cde^	1.93	nd	62.92 ^b^	6.20 ^d^	30.87 ^de^	1.30 ^cd^
White currants	26.43 ^ab^	1.27	8.77	28.05 ^ab^	10.40 ^cd^	nd	12.07 ^cd^	nd	13.02 ^cde^	nd	nd	63.23 ^b^	11.65 ^c^	25.10 ^ef^	0.92 ^de^
White gooseberries	24.70 ^abc^	nd	10.87	27.10 ^ab^	4.03 ^ef^	1.60 ^abc^	17.40^c^	nd	14.30 ^cd^	nd	nd	62.67 ^b^	5.67 ^de^	31.67 ^d^	1.22 ^cd^

^1^ Saturated Fatty Acid. ^2^ Monounsaturated Fatty Acid. ^3^ Polyunsaturated Fatty Acid. ^4^ nd = not detected. ^a,b,c,d,e,f^ Values in the column with different superscript letters are significantly different (*p* < 0.05).

## Supplementary Materials

The following are available online at https://www.mdpi.com/2304-8158/9/5/623/s1, Table S1: Area (values are expressed as arbitrary units of log10 of the peak area of the corresponding selected ion) of 70 different analytes found in the eleven investigated berries. Table S2: Polyphenolic compounds identification by HPLC-HRMS in positive polarity. Figure S1: Pie charts of the eleven berry fruits representing the percentage abundances of the individual polyphenols.

## References

[1] Manganaris: G., Goulas V., Vicente A.R., Terry L.A. (2014). Berry antioxidants: Small fruits providing large benefits. J. Sci. Food Agric..

[2] Balogh E., Hegedűs A., Stefanovits-Bányaia E. (2010). Application of and correlation among antioxidant and antiradical assays for characterizing antioxidant capacity of berries. Sci. Hortic..

[3] Namiesnik J., Vearasilp K., Kupska M., Ham K.S., Kang S.G., Park Y.K., Barasch D., Nemirovski A., Gorinstein S. (2013). Antioxidant activities and bioactive components in some berries. Eur. Food Res. Technol..

[4] Crozier A., Jaganath I.B., Clifford M.N. (2009). Dietary phenolics: Chemistry, bioavailability and effects on health. Nat. Prod. Rep..

[5] D’Archivio M., Filesi C., Varì R., Scazzocchio B., Masella R. (2010). Bioavailability of the polyphenols: Status and controversies. Int. J. Mol. Sci..

[6] Panche A.N., Diwan A.D., Chandra S.R. (2016). Flavonoids: An overview. J. Nutr. Sci..

[7] AGRION Technical Notes for Vegetables, Strawberries and Small Fruits. http://www.agrion.it/gesnew-sche.asp?GesNewId=6849&nomenu=0.

[8] Rowland L.J., Hancock J.F., Bassil N.V., Folta K.M., Kole C. (2011). Blueberry. Genetics, Genomics and Breeding of Berries.

[9] Borges G., Degeneve A., Mullen W., Crozier A. (2010). Identification of flavonoid and phenolic antioxidants in black currants, blueberries, raspberries, red currants, and cranberries. J. Agric. Food Chem..

[10] Cheng G.W., Breen P.J. (1991). Activity of phenylalanine ammonia-lyase (PAL) and concentrations of anthocyanins and phenolics in developing strawberry fruit. J. Am. Soc. Hortic. Sci..

[11] Zhishen J., Mengcheng T., Jianming W. (1999). The determination of flavonoid contents in mulberry and their scavenging effects on superoxide radicals. Food Chem..

[12] Re R., Pellegrini N., Proteggente A., Pannala A., Yang M., Rice-Evans C. (1999). Antioxidant activity applying an improved ABTS radical cation decolorization assay. Free Radic. Biol. Med..

[13] Benzie I.F., Strain J.J. (1999). Ferric reducing/antioxidant power assay: Direct measure of total antioxidant activity of biological fluids and modified version for simultaneous measurement of total antioxidant power and ascorbic acid concentration. Methods Enzymol..

[14] Amarowicz R., Estrella I., Hernández T., Troszyńska A. (2008). Antioxidant activity of extract of adzuki bean and its fractions. J. Food Lipid..

[15] Wang X., Tong H., Chen F., Gangemi J.D. (2010). Chemical characterization and antioxidant evaluation of muscadine grape pomace extract. Food Chem..

[16] Giovanelli G., Buratti S. (2009). Comparison of polyphenolic composition and antioxidant activity of wild Italian blueberries and some cultivated varieties. Food Chem..

[17] Peiretti P.G., Gai F., Tassone S. (2013). Fatty acid profile and nutritive value of quinoa (*Chenopodium quinoa* Willd.) seeds and plants at different growth stages. Anim. Feed Sci. Technol..

[18] Sariburun E., Sahin S., Demir C., Türkben C., Uylaşer V. (2010). Phenolic content and antioxidant activity of raspberry and blackberry cultivars. J. Food Sci..

[19] Dugo P., Mondello L., Errante G., Zappia G., Dugo G. (2001). Identification of anthocyanins in berries by narrow-bore high-performance liquid chromatography with electrospray ionization detection. J. Agric. Food Chem..

[20] Kähkönen M.P., Heinämäki J., Ollilainen V., Heinonen M. (2003). Berry anthocyanins: Isolation, identification and antioxidant activities. J. Sci. Food Agric..

[21] Islam T., Yu X., Singh Badwal T., Xu B. (2017). Comparative studies on phenolic profiles, antioxidant capacities and carotenoid contents of red goji berry (*Lycium barbarum*) and black goji berry (*Lycium ruthenicum*). Chem. Cent. J..

[22] Brito A., Areche C., Sepúlveda B., Kennelly E.J., Simirgiotis M.J. (2014). Anthocyanin characterization, total phenolic quantification and antioxidant features of some Chilean edible berry extracts. Molecules.

[23] Battino M., Beekwilder J., Denoyes-Rothan B., Laimer M., McDougall G.J., Mezzetti B. (2009). Bioactive compounds in berries relevant to human health. Nutr. Rev..

[24] Dragović-Uzelac V., Savić Z., Brala A., Levaj B., Bursać Kovačević D., Biško A. (2010). Evaluation of phenolic content and antioxidant capacity of blueberry cultivars (*Vaccinium corymbosum* L.) grown in the Northwest Croatia. Food Technol. Biotechnol..

[25] Cao G., Sofic E., Prior R. (1996). Antioxidant capacity of tea and common vegetables. J. Agric. Food Chem..

[26] Kähkönen M.P., Heinone M. (2003). Antioxidant activity of anthocyanins and their aglycons. J. Agric. Food Chem..

[27] You Q., Wang B., Chen F., Huang Z., Wang X., Luo P.G. (2011). Comparison of anthocyanins and phenolics in organically and conventionally grown blueberries in selected cultivars. Food Chem..

[28] Saw C.L.L., Guo Y., Yang A.Y., Paredes-Gonzalez X., Ramirez C., Pung D., Kong A.N.T. (2014). The berry constituents quercetin, kaempferol, and pterostilbene synergistically attenuate reactive oxygen species: Involvement of the Nrf2-ARE signaling pathway. Food Chem. Toxicol..

[29] Mikulic-Petkovsek M., Slatnar A., Stampar F., Veberic R. (2012). HPLC-MS^n^ identification and quantification of flavonol glycosides in 28 wild and cultivated berry species. Food Chem..

[30] Tian Y., Liimatainen J., Alanne A.L., Lindstedt A., Liu P., Sinkkonen J., Kallio H., Yang B. (2017). Phenolic compounds extracted by acidic aqueous ethanol from berries and leaves of different berry plants. Food Chem..

[31] Lee S.G., Vance T.M., Nam T.G., Kim D.O., Koo S.I., Chun O.K. (2015). Contribution of anthocyanin composition to total antioxidant capacity of berries. Plant Foods Hum. Nutr..

[32] Skenderidis P., Lampakis D., Giavasis I., Leontopoulos S., Petrotos K., Hadjichristodoulou C. (2019). Chemical properties, fatty-acid composition, and antioxidant activity of goji berry (*Lycium barbarum* L. and *Lycium chinense* Mill.) fruits. Antioxidants.

[33] Manríquez-Torres J.J., Sánchez-Franco J.A., Ramírez-Moreno E., Cruz-Cansino N.D., Ariza-Ortega J.A., Torres-Valencia J.M. (2016). Effect of thermoultrasound on the antioxidant compounds and fatty acid profile of blackberry (*Rubus fruticosus* spp.) juice. Molecules.

[34] Parry J., Su L., Moore J., Cheng Z., Luther M. (2006). Chemical compositions, antioxidant capacities, and antiproliferative activities of selected fruit seed flours. J. Agric. Food Chem..

[35] Radočaj O., Vujasinović V., Dimić E., Basić Z. (2014). Blackberry (*Rubus fruticosus* L.) and raspberry (*Rubus idaeus* L.) seed oils extracted from dried press pomace after longterm frozen storage of berries can be used as functional food ingredients. Eur. J. Lipid Sci. Technol..

[36] Van Hoed V., Barbouche I., De Clercq N., Dewettinck K., Slah M., Leber E., Verhé R. (2011). Influence of filtering of cold pressed berry seed oils on their antioxidant profile and quality characteristics. Food Chem..

[37] Yang B., Ahotupa M., Määttä P., Kallio H. (2011). Composition and antioxidative activities of supercritical CO_2_-extracted oils from seeds and soft parts of northern berries. Food Res. Int..

[38] Bushman B.S., Phillips B., Isbell T., Ou B., Crane J.M., Knapp S.J. (2004). Chemical composition of caneberry (*Rubus* spp.) seeds and oils and their antioxidant potential. J. Agric. Food Chem..

[39] Parry J., Yu L. (2004). Fatty acid content and antioxidant properties of cold-pressed black raspberry seed oil and meal. J. Food Sci..

[40] Parry J., Su L., Luther M., Zhou K., Yurawecz M.P., Whittaker P., Yu L. (2005). Fatty acid composition and antioxidant properties of cold-pressed marionberry, boysenberry, red raspberry, and blueberry seed oils. J. Agric. Food Chem..

[41] FAO (2010). Fats and Fatty Acids in Human Nutrition—Report. http://www.fao.org/3/a-i1953e.pdf.

