# Supplementary material for: Bioactive Compounds and Antioxidant Capacity of Small Berries
Source: Foods. 2020 May 13;9(5):623. doi: 10.3390/foods9050623 (PMC7278679; doi:10.3390/foods9050623)
Supplement: Supplementary file 1 [file foods-09-00623-s001.pdf]

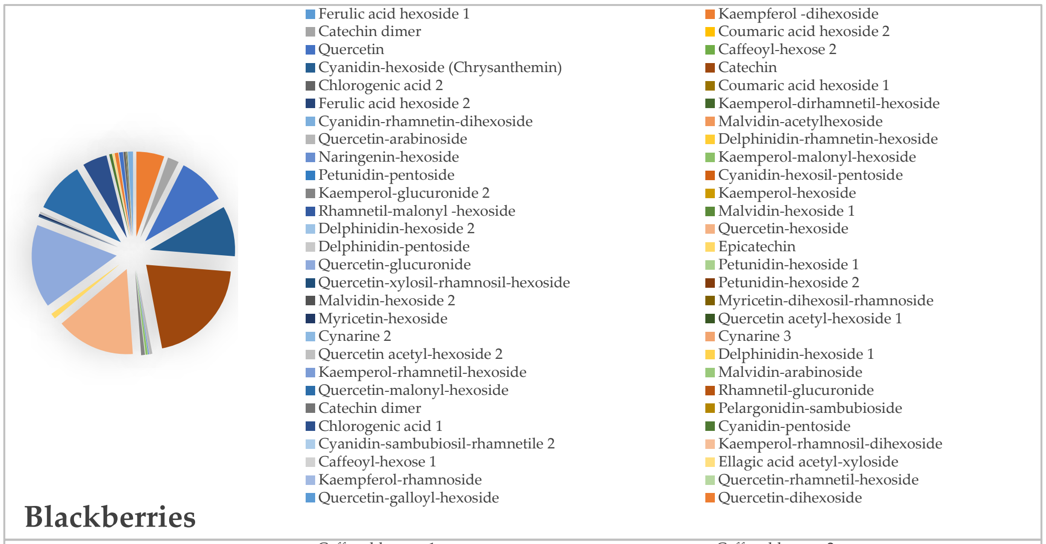
Ferulic acid hexoside 1
Catechin dimer
Quercetin
Cyanidin-hexoside (Chrysanthemin)
Chlorogenic acid 2
Ferulic acid hexoside 2
Cyanidin-rhamnetin-dihexoside
Quercetin-arabinoside
Naringenin-hexoside
Petunidin-pentoside
Kaemperol-glucuronide 2
Rhamnetil-malonyl -hexoside
Delphinidin-hexoside 2
Delphinidin-pentoside
Quercetin-glucuronide
Quercetin-xylosil-rhamnosil-hexoside
Malvidin-hexoside 2
Myricetin-hexoside
Cynarine 2
Quercetin acetyl-hexoside 2
Kaemperol-rhamnetil-hexoside
Quercetin-malonyl-hexoside
Catechin dimer
Chlorogenic acid 1
Cyanidin-sambubiosil-rhamnetile 2
Caffeoyl-hexose 1
Kaempferol-rhamnoside
Quercetin-galloyl-hexoside
Kaempferol -dihexoside
Coumaric acid hexoside 2
Caffeoyl-hexose 2
Catechin
Coumaric acid hexoside 1
Kaemperol-dirhamnetil-hexoside
Malvidin-acetylhexoside
Delphinidin-rhamnetin-hexoside
Kaemperol-malonyl-hexoside
Cyanidin-hexosil-pentoside
Kaemperol-hexoside
Malvidin-hexoside 1
Quercetin-hexoside
Epicatechin
Petunidin-hexoside 1
Petunidin-hexoside 2
Myricetin-dihexosil-rhamnoside
Quercetin acetyl-hexoside 1
Cynarine 3
Delphinidin-hexoside 1
Malvidin-arabinoside
Rhamnetil-glucuronide
Pelargonidin-sambubioside
Cyanidin-pentoside
Kaemperol-rhamnosil-dihexoside
Ellagic acid acetyl-xyloside
Quercetin-rhamnetil-hexoside
Quercetin-dihexoside
Blackberries

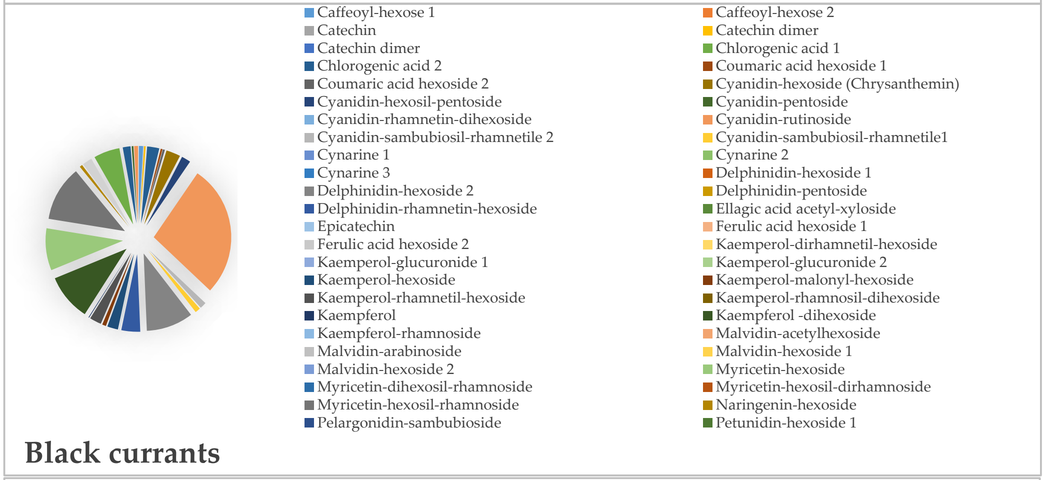
Caffeoyl-hexose 1
Catechin
Catechin dimer
Chlorogenic acid 2
Coumaric acid hexoside 2
Cyanidin-hexosil-pentoside
Cyanidin-rhamnetin-dihexoside
Cyanidin-sambubiosil-rhamnetile 2
Cynarine 1
Cynarine 3
Delphinidin-hexoside 2
Delphinidin-rhamnetin-hexoside
Epicatechin
Ferulic acid hexoside 2
Kaemperol-glucuronide 1
Kaemperol-hexoside
Kaemperol-rhamnetil-hexoside
Kaempferol
Kaempferol-rhamnoside
Malvidin-arabinoside
Malvidin-hexoside 2
Myricetin-dihexosil-rhamnoside
Myricetin-hexosil-rhamnoside
Pelargonidin-sambubioside
Caffeoyl-hexose 2
Catechin dimer
Chlorogenic acid 1
Coumaric acid hexoside 1
Cyanidin-hexoside (Chrysanthemin)
Cyanidin-pentoside
Cyanidin-rutinoside
Cyanidin-sambubiosil-rhamnetile1
Cynarine 2
Delphinidin-hexoside 1
Delphinidin-pentoside
Ellagic acid acetyl-xyloside
Ferulic acid hexoside 1
Kaemperol-dirhamnetil-hexoside
Kaemperol-glucuronide 2
Kaemperol-malonyl-hexoside
Kaemperol-rhamnosil-dihexoside
Kaempferol -dihexoside
Malvidin-acetylhexoside
Malvidin-hexoside 1
Myricetin-hexoside
Myricetin-hexosil-dirhamnoside
Naringenin-hexoside
Petunidin-hexoside 1
Black currants

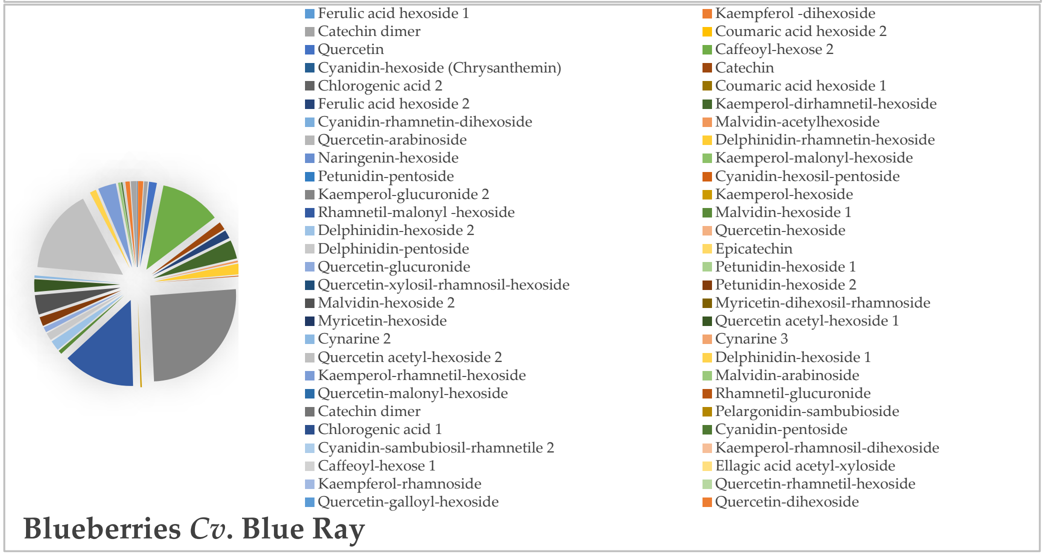
Ferulic acid hexoside 1
Catechin dimer
Quercetin
Cyanidin-hexoside (Chrysanthemin)
Chlorogenic acid 2
Ferulic acid hexoside 2
Cyanidin-rhamnetin-dihexoside
Quercetin-arabinoside
Naringenin-hexoside
Petunidin-pentoside
Kaemperol-glucuronide 2
Rhamnetil-malonyl -hexoside
Delphinidin-hexoside 2
Delphinidin-pentoside
Quercetin-glucuronide
Quercetin-xylosil-rhamnosil-hexoside
Malvidin-hexoside 2
Myricetin-hexoside
Cynarine 2
Quercetin acetyl-hexoside 2
Kaemperol-rhamnetil-hexoside
Quercetin-malonyl-hexoside
Catechin dimer
Chlorogenic acid 1
Cyanidin-sambubiosil-rhamnetile 2
Caffeoyl-hexose 1
Kaempferol-rhamnoside
Quercetin-galloyl-hexoside
Kaempferol -dihexoside
Coumaric acid hexoside 2
Caffeoyl-hexose 2
Catechin
Coumaric acid hexoside 1
Kaemperol-dirhamnetil-hexoside
Malvidin-acetylhexoside
Delphinidin-rhamnetin-hexoside
Kaemperol-malonyl-hexoside
Cyanidin-hexosil-pentoside
Kaemperol-hexoside
Malvidin-hexoside 1
Quercetin-hexoside
Epicatechin
Petunidin-hexoside 1
Petunidin-hexoside 2
Myricetin-dihexosil-rhamnoside
Quercetin acetyl-hexoside 1
Cynarine 3
Delphinidin-hexoside 1
Malvidin-arabinoside
Rhamnetil-glucuronide
Pelargonidin-sambubioside
Cyanidin-pentoside
Kaemperol-rhamnosil-dihexoside
Ellagic acid acetyl-xyloside
Quercetin-rhamnetil-hexoside
Quercetin-dihexoside
Blueberries Cv. Blue Ray

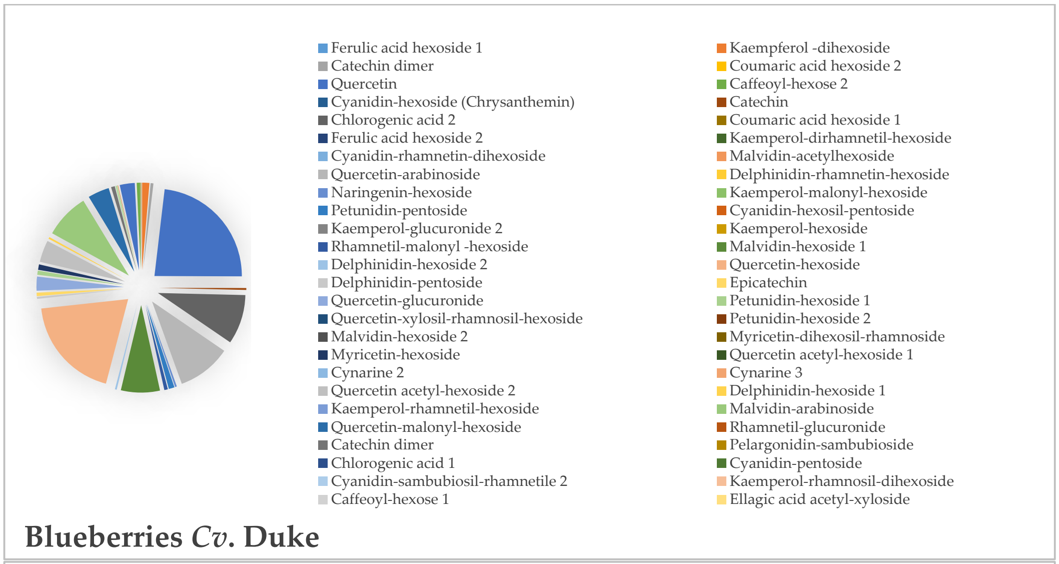
Ferulic acid hexoside 1
Catechin dimer
Quercetin
Cyanidin-hexoside (Chrysanthemin)
Chlorogenic acid 2
Ferulic acid hexoside 2
Cyanidin-rhamnetin-dihexoside
Quercetin-arabinoside
Naringenin-hexoside
Petunidin-pentoside
Kaemperol-glucuronide 2
Rhamnetil-malonyl -hexoside
Delphinidin-hexoside 2
Delphinidin-pentoside
Quercetin-glucuronide
Quercetin-xylosil-rhamnosil-hexoside
Malvidin-hexoside 2
Myricetin-hexoside
Cynarine 2
Quercetin acetyl-hexoside 2
Kaemperol-rhamnetil-hexoside
Quercetin-malonyl-hexoside
Catechin dimer
Chlorogenic acid 1
Cyanidin-sambubiosil-rhamnetile 2
Caffeoyl-hexose 1
Kaempferol -dihexoside
Coumaric acid hexoside 2
Caffeoyl-hexose 2
Catechin
Coumaric acid hexoside 1
Kaemperol-dirhamnetil-hexoside
Malvidin-acetylhexoside
Delphinidin-rhamnetin-hexoside
Kaemperol-malonyl-hexoside
Cyanidin-hexosil-pentoside
Kaemperol-hexoside
Malvidin-hexoside 1
Quercetin-hexoside
Epicatechin
Petunidin-hexoside 1
Petunidin-hexoside 2
Myricetin-dihexosil-rhamnoside
Quercetin acetyl-hexoside 1
Cynarine 3
Delphinidin-hexoside 1
Malvidin-arabinoside
Rhamnetil-glucuronide
Pelargonidin-sambubioside
Cyanidin-pentoside
Kaemperol-rhamnosil-dihexoside
Ellagic acid acetyl-xyloside
Blueberries *Cv.* Duke

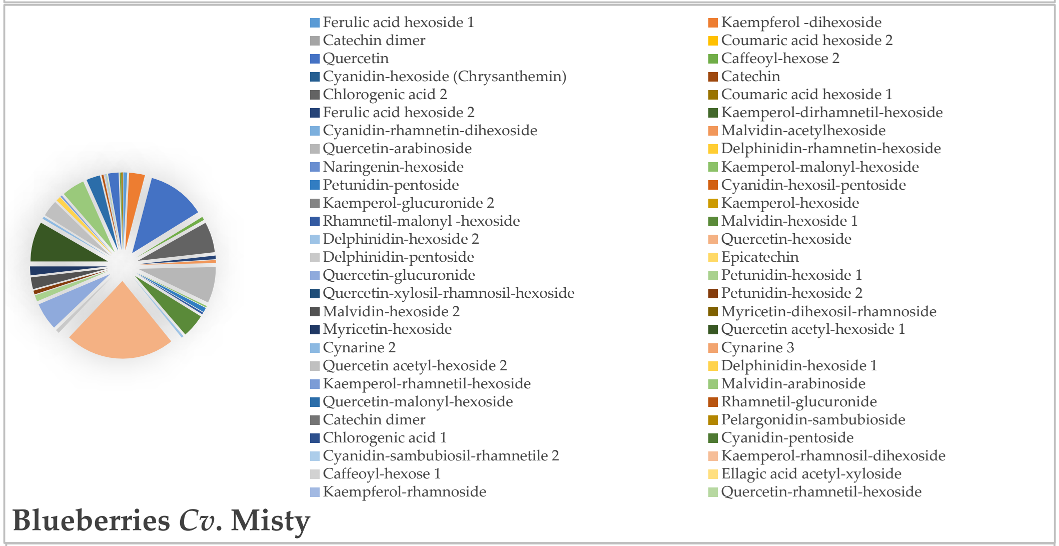
Ferulic acid hexoside 1
Catechin dimer
Quercetin
Cyanidin-hexoside (Chrysanthemin)
Chlorogenic acid 2
Ferulic acid hexoside 2
Cyanidin-rhamnetin-dihexoside
Quercetin-arabinoside
Naringenin-hexoside
Petunidin-pentoside
Kaemperol-glucuronide 2
Rhamnetil-malonyl -hexoside
Delphinidin-hexoside 2
Delphinidin-pentoside
Quercetin-glucuronide
Quercetin-xylosil-rhamnosil-hexoside
Malvidin-hexoside 2
Myricetin-hexoside
Cynarine 2
Quercetin acetyl-hexoside 2
Kaemperol-rhamnetil-hexoside
Quercetin-malonyl-hexoside
Catechin dimer
Chlorogenic acid 1
Cyanidin-sambubiosil-rhamnetile 2
Caffeoyl-hexose 1
Kaempferol-rhamnoside
Kaempferol -dihexoside
Coumaric acid hexoside 2
Caffeoyl-hexose 2
Catechin
Coumaric acid hexoside 1
Kaemperol-dirhamnetil-hexoside
Malvidin-acetylhexoside
Delphinidin-rhamnetin-hexoside
Kaemperol-malonyl-hexoside
Cyanidin-hexosil-pentoside
Kaemperol-hexoside
Malvidin-hexoside 1
Quercetin-hexoside
Epicatechin
Petunidin-hexoside 1
Petunidin-hexoside 2
Myricetin-dihexosil-rhamnoside
Quercetin acetyl-hexoside 1
Cynarine 3
Delphinidin-hexoside 1
Malvidin-arabinoside
Rhamnetil-glucuronide
Pelargonidin-sambubioside
Cyanidin-pentoside
Kaemperol-rhamnosil-dihexoside
Ellagic acid acetyl-xyloside
Quercetin-rhamnetil-hexoside
Blueberries *Cv.* Misty

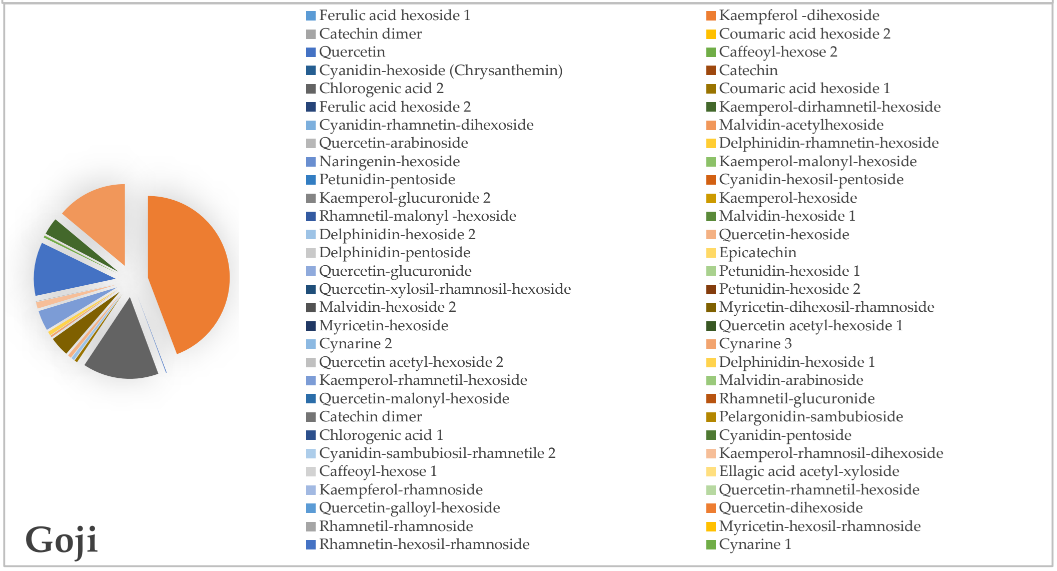
Ferulic acid hexoside 1
Catechin dimer
Quercetin
Cyanidin-hexoside (Chrysanthemin)
Chlorogenic acid 2
Ferulic acid hexoside 2
Cyanidin-rhamnetin-dihexoside
Quercetin-arabinoside
Naringenin-hexoside
Petunidin-pentoside
Kaemperol-glucuronide 2
Rhamnetil-malonyl -hexoside
Delphinidin-hexoside 2
Delphinidin-pentoside
Quercetin-glucuronide
Quercetin-xylosil-rhamnosil-hexoside
Malvidin-hexoside 2
Myricetin-hexoside
Cynarine 2
Quercetin acetyl-hexoside 2
Kaemperol-rhamnetil-hexoside
Quercetin-malonyl-hexoside
Catechin dimer
Chlorogenic acid 1
Cyanidin-sambubiosil-rhamnetile 2
Caffeoyl-hexose 1
Kaempferol-rhamnoside
Quercetin-galloyl-hexoside
Rhamnetil-rhamnoside
Rhamnetin-hexosil-rhamnoside
Kaempferol -dihexoside
Coumaric acid hexoside 2
Caffeoyl-hexose 2
Catechin
Coumaric acid hexoside 1
Kaemperol-dirhamnetil-hexoside
Malvidin-acetylhexoside
Delphinidin-rhamnetin-hexoside
Kaemperol-malonyl-hexoside
Cyanidin-hexosil-pentoside
Kaemperol-hexoside
Malvidin-hexoside 1
Quercetin-hexoside
Epicatechin
Petunidin-hexoside 1
Petunidin-hexoside 2
Myricetin-dihexosil-rhamnoside
Quercetin acetyl-hexoside 1
Cynarine 3
Delphinidin-hexoside 1
Malvidin-arabinoside
Rhamnetil-glucuronide
Pelargonidin-sambubioside
Cyanidin-pentoside
Kaemperol-rhamnosil-dihexoside
Ellagic acid acetyl-xyloside
Quercetin-rhamnetil-hexoside
Quercetin-dihexoside
Myricetin-hexosil-rhamnoside
Cynarine 1
Goji

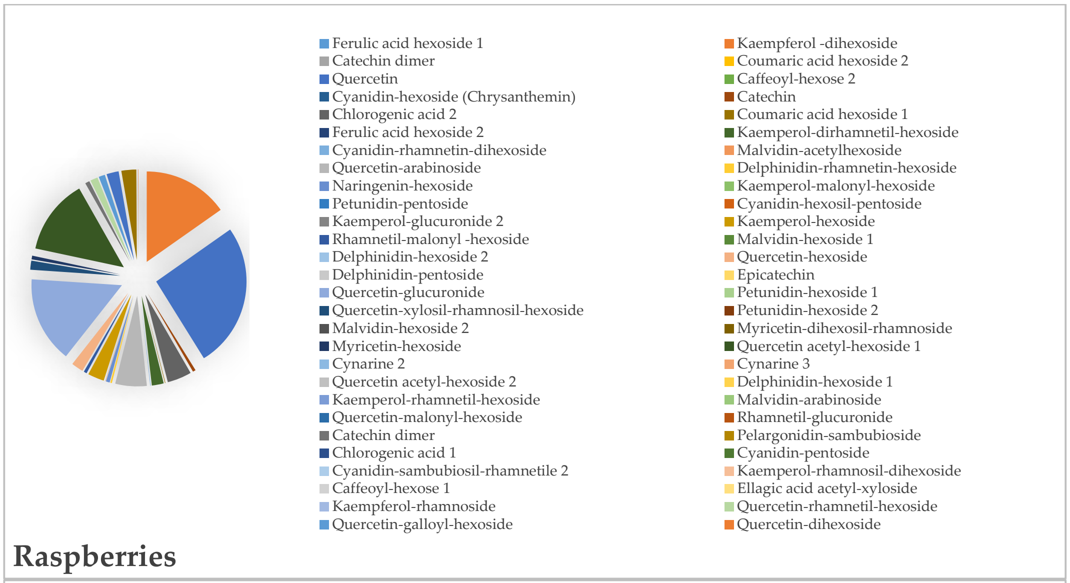
Ferulic acid hexoside 1
Catechin dimer
Quercetin
Cyanidin-hexoside (Chrysanthemin)
Chlorogenic acid 2
Ferulic acid hexoside 2
Cyanidin-rhamnetin-dihexoside
Quercetin-arabinoside
Naringenin-hexoside
Petunidin-pentoside
Kaemperol-glucuronide 2
Rhamnetil-malonyl -hexoside
Delphinidin-hexoside 2
Delphinidin-pentoside
Quercetin-glucuronide
Quercetin-xylosil-rhamnosil-hexoside
Malvidin-hexoside 2
Myricetin-hexoside
Cynarine 2
Quercetin acetyl-hexoside 2
Kaemperol-rhamnetil-hexoside
Quercetin-malonyl-hexoside
Catechin dimer
Chlorogenic acid 1
Cyanidin-sambubiosil-rhamnetile 2
Caffeoyl-hexose 1
Kaempferol-rhamnoside
Quercetin-galloyl-hexoside
Kaempferol -dihexoside
Coumaric acid hexoside 2
Caffeoyl-hexose 2
Catechin
Coumaric acid hexoside 1
Kaemperol-dirhamnetil-hexoside
Malvidin-acetylhexoside
Delphinidin-rhamnetin-hexoside
Kaemperol-malonyl-hexoside
Cyanidin-hexosil-pentoside
Kaemperol-hexoside
Malvidin-hexoside 1
Quercetin-hexoside
Epicatechin
Petunidin-hexoside 1
Petunidin-hexoside 2
Myricetin-dihexosil-rhamnoside
Quercetin acetyl-hexoside 1
Cynarine 3
Delphinidin-hexoside 1
Malvidin-arabinoside
Rhamnetil-glucuronide
Pelargonidin-sambubioside
Cyanidin-pentoside
Kaemperol-rhamnosil-dihexoside
Ellagic acid acetyl-xyloside
Quercetin-rhamnetil-hexoside
Quercetin-dihexoside
Raspberries

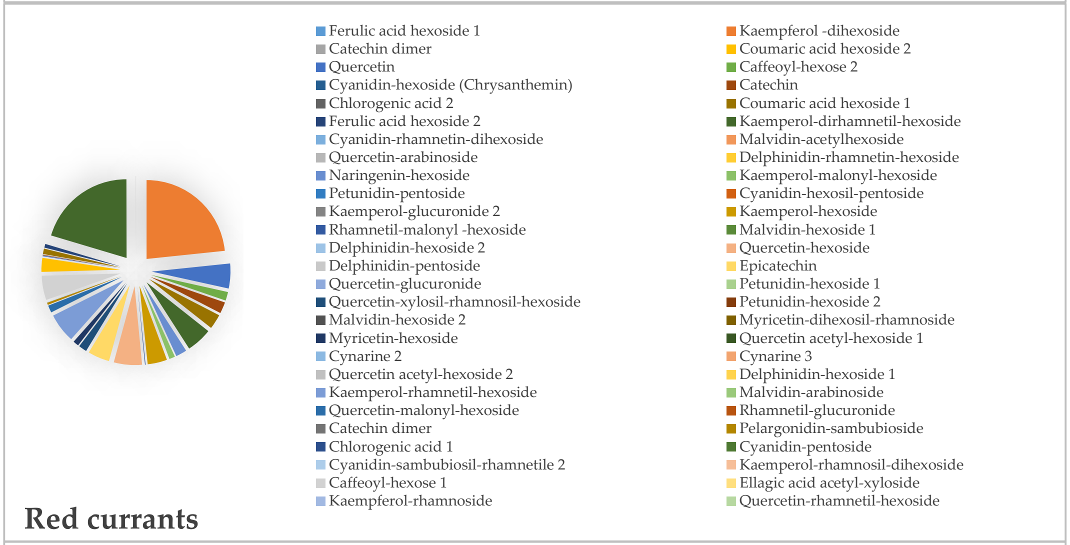
Ferulic acid hexoside 1
Catechin dimer
Quercetin
Cyanidin-hexoside (Chrysanthemin)
Chlorogenic acid 2
Ferulic acid hexoside 2
Cyanidin-rhamnetin-dihexoside
Quercetin-arabinoside
Naringenin-hexoside
Petunidin-pentoside
Kaemperol-glucuronide 2
Rhamnetil-malonyl -hexoside
Delphinidin-hexoside 2
Delphinidin-pentoside
Quercetin-glucuronide
Quercetin-xylosil-rhamnosil-hexoside
Malvidin-hexoside 2
Myricetin-hexoside
Cynarine 2
Quercetin acetyl-hexoside 2
Kaemperol-rhamnetil-hexoside
Quercetin-malonyl-hexoside
Catechin dimer
Chlorogenic acid 1
Cyanidin-sambubiosil-rhamnetile 2
Caffeoyl-hexose 1
Kaempferol-rhamnoside
Kaempferol -dihexoside
Coumaric acid hexoside 2
Caffeoyl-hexose 2
Catechin
Coumaric acid hexoside 1
Kaemperol-dirhamnetil-hexoside
Malvidin-acetylhexoside
Delphinidin-rhamnetin-hexoside
Kaemperol-malonyl-hexoside
Cyanidin-hexosil-pentoside
Kaemperol-hexoside
Malvidin-hexoside 1
Quercetin-hexoside
Epicatechin
Petunidin-hexoside 1
Petunidin-hexoside 2
Myricetin-dihexosil-rhamnoside
Quercetin acetyl-hexoside 1
Cynarine 3
Delphinidin-hexoside 1
Malvidin-arabinoside
Rhamnetil-glucuronide
Pelargonidin-sambubioside
Cyanidin-pentoside
Kaemperol-rhamnosil-dihexoside
Ellagic acid acetyl-xyloside
Quercetin-rhamnetil-hexoside
Red currants

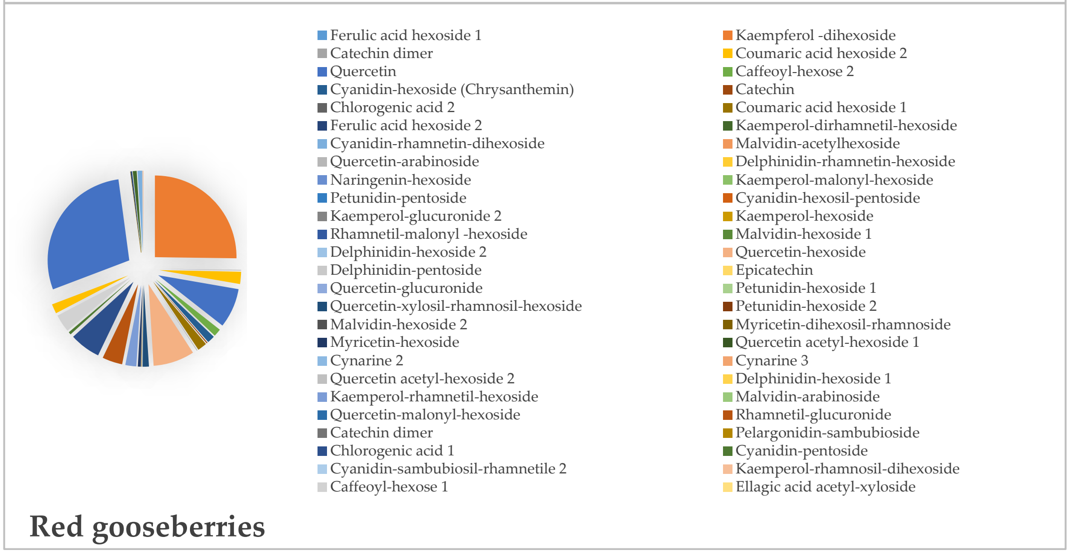
Ferulic acid hexoside 1
Catechin dimer
Quercetin
Cyanidin-hexoside (Chrysanthemin)
Chlorogenic acid 2
Ferulic acid hexoside 2
Cyanidin-rhamnetin-dihexoside
Quercetin-arabinoside
Naringenin-hexoside
Petunidin-pentoside
Kaemperol-glucuronide 2
Rhamnetil-malonyl -hexoside
Delphinidin-hexoside 2
Delphinidin-pentoside
Quercetin-glucuronide
Quercetin-xylosil-rhamnosil-hexoside
Malvidin-hexoside 2
Myricetin-hexoside
Cynarine 2
Quercetin acetyl-hexoside 2
Kaemperol-rhamnetil-hexoside
Quercetin-malonyl-hexoside
Catechin dimer
Chlorogenic acid 1
Cyanidin-sambubiosil-rhamnetile 2
Caffeoyl-hexose 1
Kaempferol -dihexoside
Coumaric acid hexoside 2
Caffeoyl-hexose 2
Catechin
Coumaric acid hexoside 1
Kaemperol-dirhamnetil-hexoside
Malvidin-acetylhexoside
Delphinidin-rhamnetin-hexoside
Kaemperol-malonyl-hexoside
Cyanidin-hexosil-pentoside
Kaemperol-hexoside
Malvidin-hexoside 1
Quercetin-hexoside
Epicatechin
Petunidin-hexoside 1
Petunidin-hexoside 2
Myricetin-dihexosil-rhamnoside
Quercetin acetyl-hexoside 1
Cynarine 3
Delphinidin-hexoside 1
Malvidin-arabinoside
Rhamnetil-glucuronide
Pelargonidin-sambubioside
Cyanidin-pentoside
Kaemperol-rhamnosil-dihexoside
Ellagic acid acetyl-xyloside
Red gooseberries

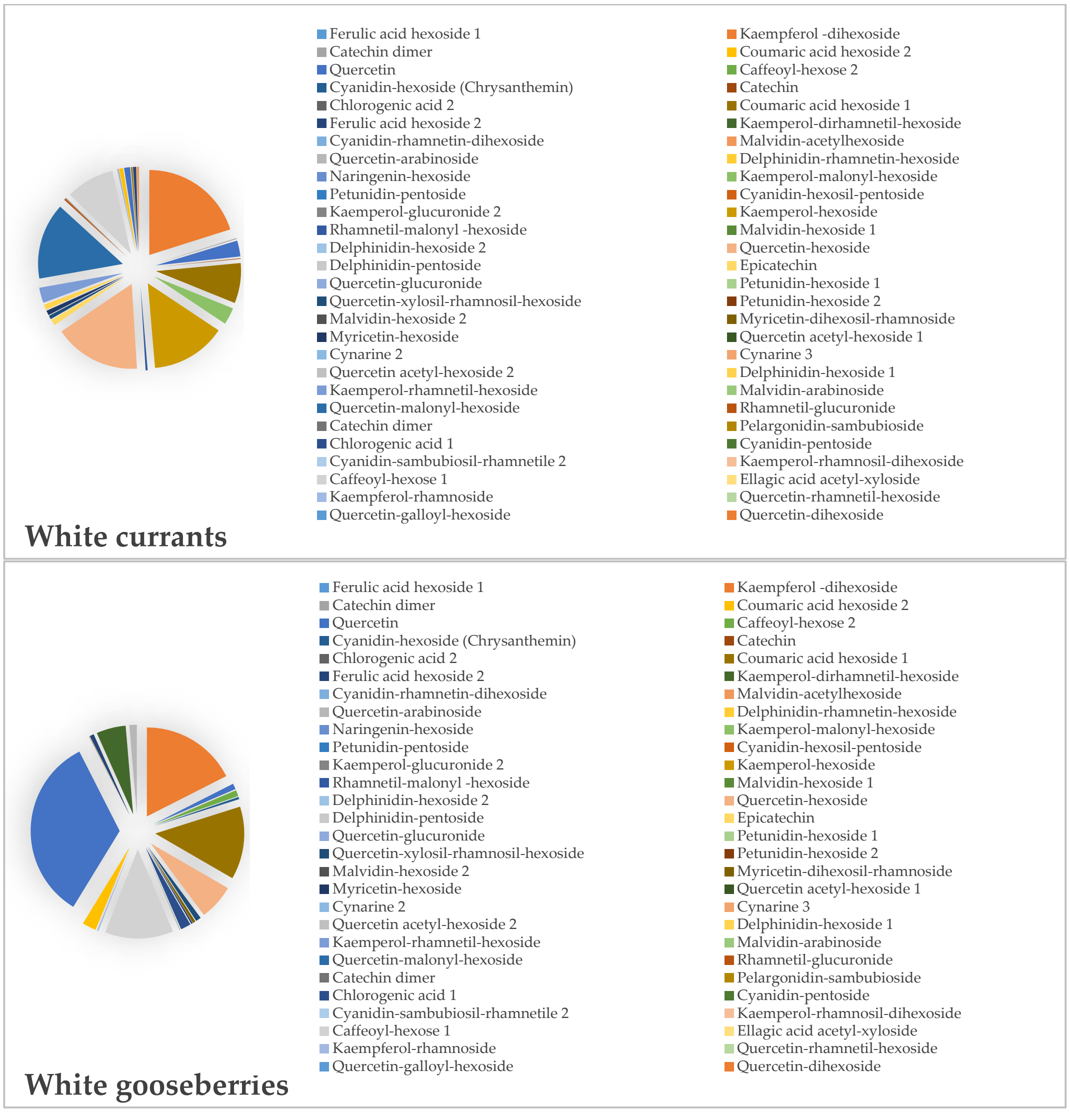

Figure S1. Pie charts of the 11 berry fruits representing the percentage abundances of the individual polyphenols.

Table S1. Area (values are expressed as arbitrary units of log_{10} of the peak area of the corresponding selection).

Analyte	*m/z*	Blackberry Loch Tay	Black currant	Blueberry Blue Ray	Blueberry Duke	Blueberry Misty	Goji	Raspberry	Red Currant	Red Gooseberry	White Currant	White Gooseberry
Kaempferol	287.0555	7.24	7.86	6.49	7.35	8.05	6.37	7.81	7.16	6.05	6.99	5.71
Epicatechin	291.0868	8.26	7.43	8.54	8.09	nd[1]	nd	nd	7.76	nd	7.58	nd
Catechin	291.0868	9.55	7.58	8.43	7.79	nd	nd	7.09	7.48	6.96	6.64	nd
Quercetin	303.0504	9.19	8.98	8.27	9.64	9.59	nd	8.80	7.82	8.56	7.99	7.01
Coumaric acid hexoside 1	327.1080	nd	8.24	nd	nd	nd	7.49	6.37	7.60	7.93	8.40	8.11
Coumaric acid hexoside 2	327.1080	nd	8.32	nd	nd	nd	nd	nd	nd	8.05	nd	nd
Coumaric acid hexoside 3	327.1080	nd	nd	nd	nd	nd	nd	nd	nd	nd	nd	nd
Coumaroyl quinic acid 1	339.1079	nd	9.15	nd	nd	7.68	nd	nd	nd	7.87	nd	nd
Coumaroyl quinic acid 2	339.1079	nd	8.46	7.88	nd	7.60	7.42	nd	nd	7.48	8.81	nd
Coumaroyl quinic acid 3	339.1079	nd	nd	7.63	nd	7.65	nd	nd	nd	7.16	8.22	nd
Caffeoyl acid hexoside 1	343.1029	nd	8.61	7.69	nd	7.96	nd	nd	7.81	8.21	8.48	8.08
Caffeoyl acid hexoside 2	343.1029	nd	nd	8.11	nd	8.35	nd	nd	7.36	7.85	nd	7.04
Chlorogenic acid 1	355.1030	8.90	nd	nd	nd	nd	nd	nd	nd	8.45	nd	7.26
Chlorogenic acid 2	355.1030	nd	9.07	10.32	10.24	10.31	8.91	8.02	nd	nd	nd	nd
Kaempferol-rhamnoside	433.1135	nd	nd	nd	nd	7.67	nd	nd	nd	nd	6.63	6.40
Apigenin-hexoside	433.1135	nd	nd	nd	nd	nd	nd	nd	nd	nd	6.74	nd
Quercetin-pentoside	435.0927	7.97	nd	8.82	9.27	9.39	nd	8.13	nd	6.82	nd	nd
Naringenin-hexoside	435.1291	7.47	8.44	7.78	7.80	nd	nd	7.22	7.47	nd	nd	nd
Kaempferol-hexoside	449.1082	nd	9.02	nd	nd	nd	nd	7.85	7.72	nd	8.67	nd
Quercetin-rhamnoside	449.1135	nd	nd	nd	nd	9.27	nd	nd	nd	nd	nd	nd
Kaempferol-glucuronide 1	463.1032	7.79	nd	nd	nd	nd	nd	nd	nd	nd	nd	nd
Kaempferol-glucuronide 2	463.1032	8.09	nd	nd	nd	nd	nd	nd	nd	nd	nd	nd
Rhamnetin-rhamnoside	463.1239	nd	nd	nd	nd	nd	6.56	nd	nd	nd	6.66	nd
Quercetin-hexoside	465.1031	9.41	9.40	9.40	9.56	9.87	7.58	7.72	7.87	8.57	8.73	7.80
Ellagic acid-acetyl-xyloside	477.0829	7.73	nd	nd	nd	nd	nd	nd	nd	nd	nd	nd
Quercetin-glucuronide	479.0825	9.43	nd	nd	8.69	nd	nd	8.57	nd	nd	nd	nd
Rhamnetin-hexoside	479.1186	nd	nd	8.80	nd	8.11	nd	nd	nd	8.26	7.04	nd
Myricetin-hexoside	481.098	nd	9.61	8.50	8.27	8.77	nd	7.20	7.18	7.43	7.46	6.39
Quercetin acetylhexoside 1	507.1301	nd	nd	8.83	nd	9.43	nd	8.51	nd	nd	nd	nd
Quercetin acetylhexoside 2	507.1301	7.81	nd	8.64	8.88	9.04	nd	nd	nd	nd	nd	nd
Cynarin 1	517.1345	nd	nd	8.30	8.10	7.82	7.33	nd	nd	nd	nd	nd
Cynarin 2	517.1345	nd	nd	nd	nd	8.19	nd	nd	nd	nd	nd	nd
Cynarin 3	517.1345	nd	nd	nd	nd	nd	7.28	nd	nd	nd	nd	nd
Kaempferol-malonyl-hexoside	535.1086	7.86	8.58	nd	nd	8.00	nd	nd	7.19	nd	8.02	nd
Quercetin-malonyl-hexoside	551.1038	9.22	8.88	8.36	8.87	8.97	nd	nd	7.28	nd	8.68	nd
Rhamnetin-malonyl-hexoside	565.1194	nd	8.24	7.47	8.10	8.10	nd	7.13	6.24	nd	7.09	nd
Procyanidin dimer 1	579.1505	8.57	8.19	8.10	8.00	nd	nd	nd	nd	6.53	6.78	nd

Procyanidin dimer 2	579.1505	nd	nd	7.95	8.12	nd	nd	7.29	nd	nd	6.60	nd
Kaempferol-rhamnosyl-hexoside	595.1665	nd	9.05	nd	nd	8.06	8.33	nd	7.89	8.00	7.97	nd
Quercetin-arabinosyl-hexoside	597.1663	7.07	nd	nd	nd	nd	nd	nd	7.01	7.11	7.29	6.88
Quercetin-arabinosyl-glucuronide	611.1459	nd	nd	nd	nd	nd	nd	nd	nd	nd	nd	nd
Quercetin-rhamnosyl-hexoside	611.1616	8.96	9.65	7.96	8.40	9.03	9.38	nd	8.48	9.06	8.82	8.23
Kaempferol-dihexoside	611.1616	nd	nd	nd	nd	nd	nd	7.51	nd	nd	nd	nd
Quercetin-galloyl-hexoside	617.1146	nd	nd	nd	nd	nd	nd	7.44	nd	nd	nd	nd
Rhamnetin-rhamnosyl-hexoside	625.1771	8.11	8.58	8.17	8.72	8.85	8.75	7.72	6.37	9.12	7.57	8.52
Quercetin-dihexoside	627.1567	8.06	nd	nd	7.23	nd	nd	nd	nd	nd	nd	nd
Myricetin-rhamnosyl-hexoside	627.1567	nd	9.73	nd	nd	nd	nd	nd	7.57	7.94	7.40	7.39
Quercetin-acetyl-arabinosyl-hexoside	639.1569	nd	nd	nd	nd	nd	nd	nd	nd	nd	nd	nd
Kaempferol-dirhamnosyl-hexoside	741.2252	nd	nd	nd	nd	nd	nd	7.71	7.83	nd	nd	nd
Quercetin-arabinosyl-rhamnosyl-hexoside	743.2025	7.39	nd	nd	nd	nd	nd	7.59	7.34	7.76	7.36	6.99
Kaempferol-rhamnosyl-dihexoside	757.2202	nd	nd	nd	nd	nd	7.83	nd	nd	nd	nd	nd
Quercetin-dirhamnosyl-hexoside	757.2202	7.23	nd	nd	nd	nd	8.26	nd	8.42	7.56	nd	7.72
Quercetin-rhamnosyl-dihexoside	773.2153	nd	nd	nd	nd	nd	8.87	6.19	nd	6.66	7.16	nd
Myricetin-dirhamnosyl-hexoside	773.2153	nd	nd	nd	nd	nd	nd	nd	5.53	nd	nd	7.11
Rhamnetin- rhamnosyl-dihexoside	787.2310	nd	nd	nd	nd	nd	8.32	nd	nd	6.74	nd	6.70
Procyanidin trimer	867.2157	nd	nd	7.42	7.42	nd	nd	7.77	nd	nd	nd	nd
Cyanidin hexoside	449.1084	9.21	8.14	nd	nd	nd	nd	nd	nd	7.83	nd	nd
Cyanidin-rhamnosyl-hexoside	581.1708	8.21	9.10	nd	nd	nd	nd	nd	nd	7.62	nd	nd
Cyanidin pentoside	419.1134	7.96	nd	nd	nd	nd	nd	nd	nd	nd	nd	nd
Cyanidin-rhamnosyl-dihexoside	757.2456	nd	nd	nd	nd	nd	nd	6.32	nd	nd	nd	nd
Delphinidin-hexoside 1	465.1032	nd	nd	7.91	7.80	8.51	nd	nd	nd	nd	nd	nd
Delphinidin-hexoside 2	465.1032	nd	8.66	7.69	7.80	8.17	nd	nd	nd	nd	nd	nd
Delphinidin-pentoside	435.0927	nd	nd	8.14	7.85	8.42	nd	nd	nd	nd	nd	nd
Petunidin-hexoside 1	479.1188	nd	nd	8.42	8.15	8.62	nd	nd	nd	nd	nd	nd

Petunidin-hexoside 2	479.1188	nd	nd	8.27	nd	8.42	nd	nd	nd	nd	nd	nd
Petunidin-pentoside	449.1082	nd	nd	8.57	8.30	8.49	nd	nd	nd	nd	nd	nd
Malvidin-hexoside 1	493.1343	nd	nd	9.67	9.13	9.20	nd	nd	nd	nd	nd	nd
Malvidin-hexoside 2	493.1343	nd	nd	nd	nd	8.90	nd	nd	nd	nd	nd	nd
Malvidin-pentoside	463.1239	nd	nd	9.46	9.19	9.19	nd	nd	nd	nd	nd	nd
Malvidin-acetyl-hexoside	535.1452	nd	nd	8.46	nd	8.32	nd	nd	nd	nd	nd	nd
Delphinidin-rhamnosyl-hexoside	611.1615	nd	9.26	nd	nd	nd	nd	nd	nd	nd	nd	nd
Cyanidin-sambubiosyl-rhamnoside 1	727.2350	nd	nd	nd	nd	nd	nd	nd	6.24	nd	nd	nd
Cyanidin-sambubiosyl-rhamnoside 2	727.2350	nd	nd	nd	nd	nd	nd	nd	5.98	nd	nd	nd

[1] nd = not detected.

Table 2. Polyphenolic compounds identification by HPLC-HRMS in positive polarity.

Analyte	[M+H] +	Occurrence in Samples	MSMS Fragments	Identification by
Kaempferol	287.0555	All the samples	137.0232, 269.2270	Standard
Epicatechin	291.0868	BB, BC, DB, LB, RC, WC [1]	139.0388, 273.0764	Standard
Catechin	291.0868	BC, BB, DB, LB, RA, RC, RG, WC	139.0388, 273.0765	Standard
Quercetin	303.0604	All the samples	257.0450, 285.0401	Standard
Coumaric acid hexoside 1	327.0868	BC, GO, RA, RC, RG, WC, WG	295.0607, 165.0547	Reference [9,31,42]
Coumaric acid hexoside 2	327.0868	BC, RG	295.0607, 165.0547	Reference [9,31,42]
Caffeoyl-hexoside 1	343.1029	BB, BC, MB, RC, RG, WC, WG	325.0714, 191.0342, 181.0861	Reference [31]
Caffeoyl-hexoside 2	343.1029	BB, MB, RC, RG, WG	325.0714, 191.0342, 181.0861	Reference [31]
Chlorogenic acid 1	355.1030	LB, RG, WG,	163.039	Reference [26]
Chlorogenic acid 2	355.1030	BB, BC, DB, GO, MB, RA	163.039	Reference [26]
Ferulic acid hexoside 1	357.1187	BB, MB	163.0391, 195.0655	MS/Reference [43]
Ferulic acid hexoside 2	357.1187	BB, MB	163.0391, 195.0655	MS
Cyanidin-pentoside	419.1134	LB, RG, WG	287.0556	Reference [9]
Kaempferol-rhamnoside	433.1135	MB, WC, WG	287.0556	MS
Delphinidin-pentoside	435.0927	BB, DB, MB	303.0505	Reference [9]
Quercetin-arabinoside	435.0927	BB, DB, LB, MB, RA, RG	303.0608	Reference [31]
Naringenin-hexoside	435.1291	BB, BC, LB, RA, RC	273.0971	MS
Kaempferol-hexoside	449.0880	BC, RA, RC, WC	287.0557	Reference [31]
Petunidin-pentoside	449.1082	BB, DB, MB	317.0661	Reference [9,44]
Cyanidin-hexoside (Chrysanthemin)	449.1084	BC, LB, RG, WG	287.0555	Reference [9]
Kaempferol-glucuronide 1	463.1032	LB	287.065	MS
Kaempferol-glucuronide 2	463.1032	LB	287.065	MS
Malvidin-arabinoside	463.1239	BB, DB, MB	331.0818	Reference [9]
Rhamnetin-rhamnoside	463.1239	GO, WC	317.2076	MS
Quercetin-hexoside	465.1031	All the samples	303.0608	Reference [31,45]
Delphinidin-hexoside 1	465.1032	BB, DB, GO, MB, WC	303.0506	Reference [9]
Delphinidin-hexoside 2	465.1032	BB, BC, DB, GO, MB	303.0506	Reference [9]
Ellagic acid acetyl-xyloside	477.0829	LB	301.0461, 179.0054	MS
Quercetin-glucuronide	479.0825	LB	303.0608	Reference [31,44]
Rhamnetin-glucuronide	479.1186	BB, MB, RG, WC	317.2076	Reference [31,44]
Petunidin-hexoside 1	479.1188	BB, DB, MB	317.0662	Reference [26]
Petunidin-hexoside 2	479.1188	BB, MB	317.0662	Reference [26]
Myricetin-hexoside	481.0980	BB, BC, DB, LB, MB, RA, RC, RG, WC, WG	319.0572	Reference [31]
Malvidin-hexoside 1	493.1343	BB, DB, MB	331.0818	MS
Malvidin-hexoside 2	493.1343	MB	331.0818	MS
Quercetin-acetyl-hexoside 1	507.1303	BB, MB, RA	303.0505	Reference [46]
Quercetin-acetyl-hexoside 2	507.1303	BB, DB, LB, MB	303.0506	Reference [46]
Cynarine 1	517.1345	BB, DB, GO, MB	499.1238	MS
Cynarine 2	517.1345	MB	499.1238	MS
Cynarine 3	517.1345	GO	499.1238	MS
Kaempferol-malonyl-hexoside	535.1086	BC, LB, MB, RC, WC	287.0650, 491.1348	MS
Malvidin-acetyl-hexoside	535.1452	BB, MB	331.082	Reference [26]
Quercetin-malonyl-hexoside	551.1220	BB, BC, DB, LB, MB, RC, WC	303.0607	Reference [31]
Rhamnetin-malonyl-hexoside	565.1194	BB, BC, DB, LB, MB, RC, WC	317.2075	MS
Pelargonidin-sambubioside	565.176	RC	271.243	MS
Procyanidin dimer	579.1505	BB, BC, DB, LB, RG, WC	291.087, 427.1169	MS
Procyanidin dimer	579.1505	BB, DB, RA, WC	291.087, 427.1169	MS
Cyanidin-hexosyl-pentoside	581.1708	BC, RA	449.0394, 419.1182	MS
Cyanidin-rutinoside	595.1664	BC, LB, RA, RG,	287.0556	MS
Kaempferol-rhamnetin-hexoside	595.1665	GO, MB, RG, WC	287.0650, 449.1230	MS

Quercetin-xilosil-hexoside	597.1663	LB, RC, RG, WC, WG	303.0605, 465.0927, 435.1341	MS
Delphinidin-rhamnetin-hexoside	611.1514	BC, RA	303.0554, 465.1079	MS
Kaempferol-dihexoside	611.1616	All the samples	287.0652, 449.123	Reference [47]
Quercetin-rhamnetin-hexoside	611.1616	BB, DB, RA, MB	303.0608, 465.1187	MS/Reference [44]
Quercetin-galloyl-hexoside	617.1146	RA	303.0605, 465.1028	MS
Rhamnetin-hexosyl-rhamnoside	625.1771	All the samples	317.0663, 479.1186	Reference [31]
Quercetin-dihexoside	627.1567	DB, LB	303.0605	Reference [31]
Myricetin-hexosyl-rhamnoside	627.1567	BC, RC, RG, WC, WG	319.0570, 465.103	MS
Syringetin-rutinoside 1	655.2036	MB	357.0975, 623.1766, 389.1237	Reference [31,48]
Syringetin-rutinoside 2	655.2036	MB	357.0975, 623.1766, 389.1237	Reference [31,48]
Cyanidin-sambubiosyl-rhamnetin 1	727.2350	BC, RC	287.0555, 433.3021	MS
Cyanidin-sambubiosyl-rhamnetin 2	727.2350	BC, RC	287.0555, 433.3020	MS
Kaempferol-dirhamnetil-hexoside	741.2253	RA, RC	287.0557, 595.1662, 449.1082	MS
Quercetin-xilosil-rhamnosyl-hexoside	743.2025	LB, RA, RC, RG, WC, WG	303.0506, 611.1613, 465.1031	MS
Kaempferol-rhamnosyl-dihexoside	757.2202	GO	449.1080, 595.1661, 287.0556	MS
Quercetin-dirhamnosyl-hexoside	757.2202	GO, LB, RC, RG, WG	611.1614, 303.0506	Reference [31]
Cyanidin-rhamnetin-dihexoside	757.2456	RA	595.1930, 433.1404, 287.0556	MS
Quercetin-rhamnosyl-dihexoside	773.2153	GO, RA, RG, WC	465.1030, 627.1561, 611.1612, 303.0506	Reference [31]
Myricetin-hexosyl-dirhamnoside	773.2153	RC, WG	319.0455, 627.1563	MS
Myricetin-dihexosyl-rhamnoside	787.2310	GO, RG, WG	479.1186, 641.1716, 317.0662	MS

[1]BB = Blueberries *Cv.* Blue Ray, BC = Black Currant, DB = Blueberries *Cv.* Duke, GO = Goij, LB = Blackberry *Cv.* Loch Tay, MB = Blueberries *Cv.* Misty, MS = Mass Spectrometry, RA = Raspberries, RC = Red Currant, RG = Red Gooseberry, WC = White Currant, WG = White gooseberry.

REFERENCE

42 Skrovankova, S.; Sumczynski, D.; Mlcek, J.; Jurikova, T.; Sochor, J. Bioactive compounds and antioxidant activity in different types of berries. *Int. J. Mol. Sci.* **2015**, *16*, 24673–24706, doi:0.3390/ijms161024673.

43 Ieri, F.; Innocenti, M.; Possieri, L.; Gallori, S.; Mulinacci, N. Phenolic composition of “bud extracts” of *Ribes nigrum* L., *Rosa canina* L. and *Tilia tomentosa* M. *J. Pharm. Biomed. Anal.* **2015**, *115*, 1–9, doi:10.1016/j.jpba.2015.06.004.

44 Ancillotti, C.; Ciofi, L.; Pucci, D.; Sagona, E.; Giordani, E.; Biricolti, S.; Gori, M.; Petrucci, W.A.; Giardi, F.; Bartoletti R.; et al. Polyphenolic profiles and antioxidant and antiradical activity of Italian berries from *Vaccinium myrtillus* L. and *Vaccinium uliginosum* L. subsp. *gaultherioides* (Bigelow) S.B. Young. *Food Chem.* **2016**, *204*, 176–184, doi:10.1016/j.foodchem.2016.02.106.

45 Liu, P.; Kallio, H.; Yang, B. Phenolic compounds in hawthorn (*Crataegus grayana*) fruits and leaves and changes during fruit ripening. *J. Agric. Food Chem.* **2011**, *59*, 11141–11149, doi:10.1021/jf202465u.

46 Jang, G.H.; Kim, H.W.; Lee, M.K.; Jeong, S.Y.; Bak, A.R.; Lee, D.J.; Kim, J.B. Characterization and quantification of flavonoid glycosides in the Prunus genus by UPLC-DAD-QTOF/MS. *Saudi J. Biol. Sci.* **2018**, *25*, 1622–1631, doi:10.1016/j.sjbs.2016.08.001.

47 Lavola, A.; Karjalainen, R.; Julkunen-Tiitto, R. Bioactive polyphenols in leaves, stems, and berries of Saskatoon (*Amelanchier alnifolia* Nutt.) cultivars. *J. Agric. Food Chem.* **2012**, *60*, 1020–1027, doi:10.1021/jf204056s.

48 Fang, R.; Veitch, N.C.; Kite, G.C.; Porter, E.A.; Simmonds, M.S.J. Enhanced profiling of flavonol glycosides in the fruits of sea buckthorn (*Hippophae rhamnoides*). *J. Agric. Food Chem.* **2013**, *61*, 3868–3875, doi:10.1021/jf304604v.
